# Identifying Glucose Metabolism Status in Nondiabetic Japanese Adults Using Machine Learning Model with Simple Questionnaire

**DOI:** 10.1155/2022/1026121

**Published:** 2022-09-09

**Authors:** Tomoki Uchida, Takeshi Kanamori, Takanori Teramoto, Yuji Nonaka, Hiroki Tanaka, Satoshi Nakamura, Norihito Murayama

**Affiliations:** ^1^Suntory Global Innovation Center Limited, Research Institute, 8-1-1 Seikadai, Seika-cho, Soraku-gun, Kyoto 619-0284, Japan; ^2^Graduate School of Science and Technology, Nara Institute of Science and Technology, 8916-5 Takayama-cho, Ikoma, Nara 630-0192, Japan; ^3^Research Planning Division, Suntory Holdings Limited, 2-3-3 Daiba, Minato-ku, Tokyo 135-8631, Japan; ^4^Data Science Center, Nara Institute of Science and Technology, 8916-5 Takayama-cho, Ikoma, Nara 630-0192, Japan

## Abstract

We aimed to identify the glucose metabolism statuses of nondiabetic Japanese adults using a machine learning model with a questionnaire. In this cross-sectional study, Japanese adults (aged 20–64 years) from Tokyo and surrounding areas were recruited. Participants underwent an oral glucose tolerance test (OGTT) and completed a questionnaire regarding lifestyle and physical characteristics. They were classified into four glycometabolic categories based on the OGTT results: category 1: best glucose metabolism, category 2: low insulin sensitivity, category 3: low insulin secretion, and category 4: combined characteristics of categories 2 and 3. A total of 977 individuals were included; the ratios of participants in categories 1, 2, 3, and 4 were 46%, 21%, 14%, and 19%, respectively. Machine learning models (decision tree, support vector machine, random forest, and XGBoost) were developed for identifying the glycometabolic category using questionnaire responses. Then, the top 10 most important variables in the random forest model were selected, and another random forest model was developed using these variables. Its areas under the receiver operating characteristic curve (AUCs) to classify category 1 and the others, category 2 and the others, category 3 and the others, and category 4 and the others were 0.68 (95% confidence intervals: 0.62–0.75), 0.66 (0.58–0.73), 0.61 (0.51–0.70), and 0.70 (0.62–0.77). For external validation of the model, the same dataset of 452 Japanese adults in Hokkaido was obtained. The AUCs to classify categories 1, 2, 3, and 4 and the others were 0.66 (0.61–0.71), 0.57 (0.51–0.62), 0.60 (0.50–0.69), and 0.64 (0.57–0.71). In conclusion, our model could identify the glucose metabolism status using only 10 factors of lifestyle and physical characteristics. This model may help the larger general population without diabetes to understand their glucose metabolism status and encourage lifestyle improvement to prevent diabetes.

## 1. Introduction

The number of people with diabetes is increasing globally. 463 million people worldwide had diabetes as of 2019, and this number is estimated to rise to 700 million by 2045 [1]. Lifestyle modifications and pharmacological interventions can reduce the risk of developing diabetes in the future [2–6]. Decreased insulin sensitivity and impaired insulin secretion play a major role in the pathogenesis of diabetes [7, 8]. Currently available data suggest that impaired insulin secretion is primarily due to genetic factors and aging, whereas decreased insulin sensitivity is primarily due to obesity and low muscle mass [9–12]. Therefore, it is important for individuals without diabetes to understand their glucose metabolism status, i.e., insulin sensitivity and secretion, and to take appropriate measures for preventing diabetes.

Oral glucose tolerance test (OGTT) is a standard method for measuring glucose metabolism and diagnosing diabetes and prediabetes [13]. In this test, a patient is loaded with glucose solution, and multiple blood samples are drawn to measure changes in blood glucose levels. Therefore, it is rarely performed on individuals without diabetes. Thus, simpler tools have been developed to screen for prediabetes more easily than the OGTT.

Table 1 shows a review of the recent and important studies on prediabetes screening tools. De Silva et al. [14] identified predictors of individuals with high fasting plasma glucose level (FPG), high hemoglobin A1c (HbA1c), or high plasma glucose level during OGTT. Combined use of the feature selection and machine learning including random forests (RF), gradient boosting machine (GBM), logistic regression (LR), and artificial neural network (ANN) selected 25 socioeconomic, clinical, and biochemical factors. They used the dataset from the National Health and Nutrition Examination Survey (NHANES). The predictors were suitable when existing survey information was available. However, it may incur effort and cost to obtain new survey dataset for screening. In a similar technique in other fields, Chang et al. [15] developed an efficient method for classifying neonatal cry. They used RF for selecting the highly discriminative acoustic features and then classified neonatal cry using the extreme gradient boosting-powered grouped-support-vector network. The combination of variable selection and machine learning model resulted in high classification accuracy. Birk et al. [16] developed a tool for screening individuals with high FPG using global diet quality score (GDQS) and lifestyle questionnaire responses. In this study, RF, generalized linear mixed model (GLMM), least absolute shrinkage and selection operator (LASSO), and elastic net (EN) were used. They showed that dietary factors were important for prediabetes screening. However, well-trained interviewers were needed to obtain dietary information such as GDSQ. Abbas et al. [17] reported a risk score for screening individuals with high HbA1c. They used only noninvasively measured factors, including age, sex, body mass index (BMI), waist circumference, and blood pressure. The algorithms utilized included RF, GBM, XGBoost (XGB), LR, and deep learning (DL). Moreover, Dong et al. [18] developed a risk assessment model to detect individuals with high FPG or high HbA1c. Eight noninvasively measured risk factors, including age, BMI, waist-to-hip ratio, systolic blood pressure, waist circumference, sleep duration, smoking, and recreational activity time were selected. The XGB model showed superior performance than the LR model. The study characteristically used indicators of sleep and exercise in addition to clinical factors. However, in these two studies, individuals with high blood glucose level after glucose loading were not included in the screening target. In addition, some of the factors could not be evaluated on the spot and may require laboratory data. Tian et al. [19] developed a risk score for prediabetes and diabetes using questionnaires and blood test results using LR model. Age, sex, BMI, smoking, FPG, fasting plasma triglyceride level, and history of high FPG were used as factors. However, research participants were limited to the staff of an oil field in China. In addition, invasive measurement factors were required for screening. Shen et al. [20] analyzed the association between dietary patterns and prediabetes risk using the validated semiquantitative food frequency questionnaire (SQFFQ). Multivariate logistic regression analysis showed that the dietary Western pattern score and grains-vegetables pattern score predicted prediabetes risk. However, clinical and anthropometric measurements were also needed for adjustment. In addition, well-trained interviewers were needed to obtain dietary information. In these previous studies, machine learning models were used more often than ANN, which is less interpretable, to analyze the relationship between those factors and the pathogenesis of prediabetes.

In this study, we aimed to develop a machine learning model to identify glucose metabolism status in nondiabetic adults. The present study has two unique contributions. First, the factors of the model include only lifestyle and physical information that can be answered on the spot. Because invasive measurement factors or several factors are not needed, it can be easily and widely used by general population. Second, we identified glucose metabolism status rather than prediabetes. No tools have been reported to determine glucose metabolism status in nondiabetic individuals. Previously, we classified glucose metabolism status of nondiabetic individuals into four different categories based on OGTT results [21]. Each category had clearly different characteristics of insulin sensitivity and insulin secretion: category 1: best glucose metabolism, category 2: low insulin sensitivity, category 3: low insulin secretion, and category 4: combined characteristics of both categories 2 and 3. In this study, we develop a model to identify these four categories of glucose metabolism status.

## 2. Materials and Methods

### 2.1. Study Design

In this cross-sectional study, we recruited Japanese adults without diabetes aged 20–64 years in Tokyo and the surrounding area in 2019. Those with cardiovascular disorders, liver disorders, and kidney disorders and those taking medication, pregnant women, and lactating women were excluded. Diabetes was defined as a fasting plasma glucose level ≥126 mg/dL, 120-min postload plasma glucose level during the OGTT (120 mPG) ≥200 mg/dL, and/or the use of antidiabetic medications [13]. Participants underwent height and weight measurements and 75 g OGTT. Blood sampling in the OGTT was performed before glucose loading and 30, 60, 90, and 120 minutes after glucose loading. Participants also completed a questionnaire on lifestyle and physical characteristics. Those who did not answer the questionnaire and those who answered less than 90% of the questionnaire were excluded from the analysis. A total of 977 participants were suitable for the study. For external verification data, we recruited Japanese adults without diabetes aged 20–64 years in Hokkaido, Japan, in 2021. The selection and exclusion criteria were the same. The same examinations and questionnaire were conducted on them. A total of 452 participants were suitable for the study. The Matsuda index and homeostatic model assessment-insulin resistance (HOMA-IR) were calculated as indices to reflect insulin sensitivity. The Matsuda index was calculated as follows: 10,000/[square root of (fasting glucose × fasting insulin) × (mean glucose × mean insulin during the OGTT)] [22].

These two studies were conducted in accordance with the guidelines in the Helsinki Declaration (as revised by the Fortaleza General Meeting of the World Medical Association, Brazil, 2013). All participants provided written informed consent. These two studies complied with the Ethical Guidelines for Medical Research Involving Human Subjects (2014 Ministry of Education, Culture, Sports, Science and Technology and the Ministry of Health, Government of Japan, Labour and Welfare Ministerial notification No. 3). All procedures were approved by the ethics committees of Nihonbashi Egawa Clinic or Fukuhara Clinic Clinical trial review committee. They were registered at the University Hospital Medical Information Network-Clinical Trials Registry (UMIN-CTR) (registration number: UMIN000037674, UMIN000044484).

### 2.2. Classification of Glycometabolic Category

Participants were classified into the four glycometabolic categories based on plasma glucose levels and Matsuda index during the OGTT. The four categories were the objective variables of the models in this study. The classification criteria were as follows: condition A: 30 mPG <157 mg/dL and condition B: 120 mPG <126 mg/dL and Matsuda index>4.97, category 1 satisfies conditions A and B, category 2 satisfies condition A but not condition B, category 3 satisfies condition B but not condition A, and category 4 satisfies neither condition A nor condition B. The rationale for the categorization and characteristics of each category were explained in our previous study [21].

### 2.3. Model Development

For the explanatory variables, we obtained a dataset that included age, sex, height, BMI, and questionnaire responses. The questionnaire consisted of 309 questions that did not require clinical examination data and could be answered easily on the spot (Supplementary figure 1). The topics of the questions included exercise habits, sleep habits, drowsiness, dietary habits, drinking, family history, constitution, physical condition, and lifestyle. As a pretreatment for the analysis, missing answers were replaced with the mode. Questions with answers in the nominal variable were split, and each answer was converted to a dummy variable. To identify multicollinearity, the correlations between all variables were evaluated. To ensure that no variables had Spearman's correlation coefficients greater than 0.7, if two variables had a correlation coefficient greater than 0.7, one of them was excluded. Before inputting the data into the support vector machine, training and testing datasets were standardized (mean of 0 and variance of 1), respectively.

**Figure 1:**
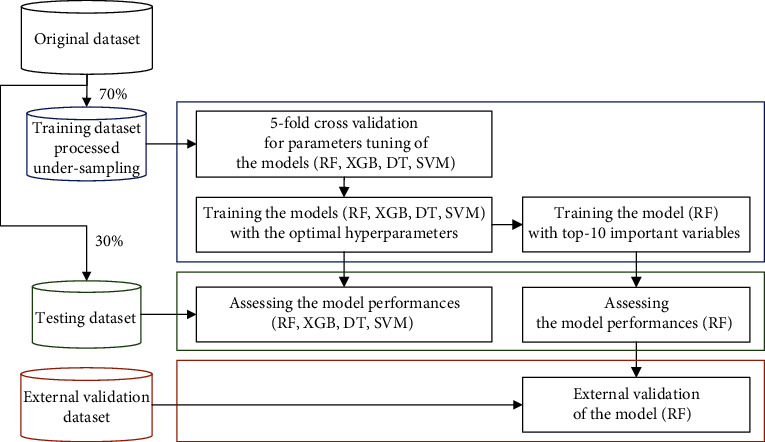
Training, testing, and validation processes of the models. Abbreviations: RF: random forest; XGB: XGBoost; DT: decision tree; SVM: support vector machine.

Four different models (decision tree, support vector machine, random forest, and XGBoost) were developed. We used these models rather than deep learning models to interpret the importance of variables and to develop a simpler model by narrowing down the variables. The rpart package of R ver. 4.1.0 was used for the decision tree. The tuned hyperparameters were the minimum number of observations in a node and maximum depth of trees. The randomForest package of R ver. 4.1.0 was used for random forest. The tuned hyperparameters were the number of variables randomly sampled at each tree, minimum size of terminal nodes, and number of trees to grow. The kernlab package of R ver. 4.1.0 was used for the support vector machine. The tuned hyperparameter was the cost of constraints violation. The kernel function was set to linear kernel. The xgboost package of R ver. 4.1.0 was used for XGBoost. The tuned hyperparameters were the subsample ratio to all variables at each tree, maximum depth of trees, and learning rate.  Figure 1 shows the training, testing, and validation processes of the models. The original dataset was randomly split into training (70%) and testing (30%) datasets. The training dataset was undersampled because four categories were imbalanced. 5-fold cross-validation was performed to find the optimal hyperparameters using the training dataset. Then, the models with the optimal hyperparameters were trained using the training dataset. The model performances were assessed using the testing dataset.

In addition, the top 10 most important variables in the random forest model were selected. The importance of each variable was assessed by the mean decrease in Gini coefficient. It is the mean of the total decrease in node impurity by a variable, weighted by the proportion of samples reaching that node in each individual decision tree in the random forest. Another random forest model with only these 10 variables as explanatory variables was trained using the training dataset. The model performances were assessed using the testing dataset and verified using the external verification dataset.

### 2.4. Model Performances

The testing dataset was used to assess the model performances. The performances were considered based on the areas under the receiver operating characteristic curve (AUCs) for classifying category 1 and the others, category 2 and the others, category 3 and the others, category 4 and the others, and the mean of these AUCs. The 95% confidence interval of the AUC was computed with 2,000 stratified bootstrap replicates. We used Delong's method to calculate *p* values to compare the AUCs [23]. In addition, the threshold was adjusted so that the sensitivity for detecting categories 2, 3, and 4 (impaired glucose metabolism groups) was as close to 0.7 as possible. Then, the specificity was evaluated at that threshold.

### 2.5. Other Statistical Analysis

The characteristics of each glycometabolic category and the OGTT values were compared using analysis of variance (ANOVA) with Dunnett's test for multiple comparisons [24]. For the insulinogenic index and disposition index, outliers were excluded by Smirnov–Grubbs test. Spearman's correlation test was used to calculate the relationships between the variables. A *p* value <0.05 was considered to indicate statistical significance. Statistical analysis was performed using the statistical software package R ver. 4.1.0 (R Foundation for Statistical Computing, Vienna, Austria).

## 3. Results

### 3.1. Participant Characteristics

Of the total of 977 eligible participants in the original dataset, the glycometabolic categories 1, 2, 3, and 4 accounted for 46% (*n* = 448), 21% (*n* = 206), 14% (*n* = 133), and 19% (*n* = 190), respectively (Table 2). Regarding the age, categories 3 and 4 were significantly higher than category 1. Regarding the BMI, categories 2 and 4 were significantly higher than category 1. The questionnaire answers were obtained from 977 participants. None of the subjects had more than 1% of missing answers. Missing answers were replaced with the mode. Nominal variable answers were split and converted to dummy variables. If two variables had a correlation coefficient greater than 0.7, one of them was excluded. Supplementary Table 1 shows the characteristics of the preprocessed 279 questionnaire answers in each category.

### 3.2. Model Performances

Table 3 lists the performances of the models. The random forest model had the highest performance among the models in terms of AUCs. Its AUCs (95% confidence intervals) to classify category 1 and the others, category 2 and the others, category 3 and the others, and category 4 and the others were 0.69 (0.63–0.75), 0.68 (0.61–0.75), 0.63 (0.55-0.72), and 0.67 (0.59-0.74). However, there was no statistically significant difference from the AUCs of the other models. Its specificity was 0.46 when the threshold was adjusted so that the detection sensitivities of categories 2, 3, and 4 (impaired glucose metabolism groups) were set to 0.7.

### 3.3. Model Performance Using 10 Variables

**Figure 2:**
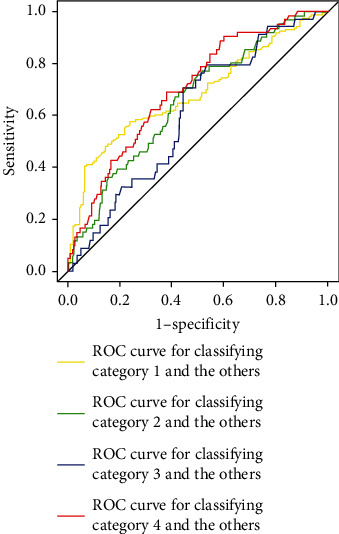
Receiver operating characteristic (ROC) curves of the random forest model using the ten variables.

In the random forest model, the top 10 most important variables were age, height, BMI, and the following questions: “Do you wake up in the middle of the night,” “Which do you usually eat: rice or bread,” “Frequency of tea intake per week at lunch,” “Do you wake up late on nonworking day,” “Frequency of mobile phone and tablet computer use at bedtime,” “Frequency of soup intake,” and “Frequency of toothbrush replacement.” Then, another random forest model was developed using only these variables.  Table 4 shows the performance of the model. Its AUCs (95% confidence intervals) to classify category 1 and others, category 2 and others, category 3 and others, and category 4 and others were 0.68 (0.62–0.75), 0.66 (0.58–0.73), 0.61 (0.51–0.70), and 0.70 (0.62–0.77), respectively. The AUC to classify category 1 and others was not significantly different from that of the previous random forest model shown in Table 3 (*p* value was 0.86). Moreover, its AUCs to classify category 2 and others, category 3 and others, and category 4 and others were not significantly different from those of the previous random forest model shown in Table 3 (*p* values were 0.33, 0.51, and 0.11).  Figure 2 shows the receiver operating characteristic (ROC) curves of the model. The AUC for classifying category 4 and others was the highest among the AUCs for classifying each category.  Table 5 shows the importance of the 10 variables in the model.

### 3.4. Model Performance in the External Validation

**Figure 3:**
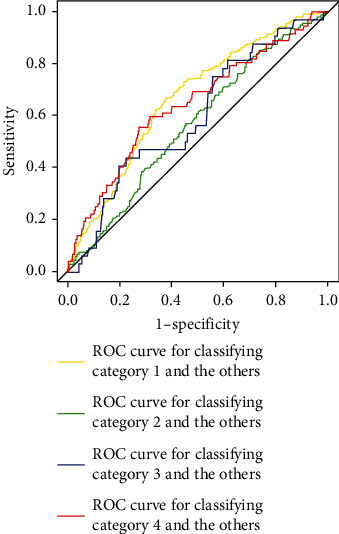
Receiver operating characteristic curves of the random forest model using the ten variables in the external validation.

Of the total of 452 eligible participants in the external validation dataset, the glycometabolic categories 1, 2, 3, and 4 accounted for 47% (*n* = 213), 30% (*n* = 135), 7% (*n* = 32), and 16% (*n* = 72), respectively (Supplementary Table 2). The questionnaire answers were obtained from the participants (Supplementary Table 3). The performance of the previous random forest model using ten variables was verified using the external validation dataset. Its AUCs (95% confidence intervals) to classify category 1 and others, category 2 and others, category 3 and others, and category 4 and others were 0.66 (0.61–0.71), 0.57 (0.51–0.62), 0.60 (0.50–0.69), and 0.64 (0.57–0.71) (Table 6). Its AUC for classifying category 1 and others was not significantly different from that of the testing dataset shown in Table 4 (*p* value was 0.63). Moreover, its AUCs for classifying category 2 and others, category 3 and others, and category 4 and others were not significantly different from those of the testing dataset shown in Table 4 (*p* values were 0.06, 0.26, and 0.89).  Figure 3 shows the receiver operating characteristic (ROC) curves of the model. The AUC for classifying category 1 and the others was the highest among the AUCs for classifying each category.

## 4. Discussion

In this study, we identified the glucose metabolism status of nondiabetic Japanese adults using a questionnaire. This model had two unique features. The first is it identifies the glucose metabolism status of individuals without diabetes. In our previous study, we classified the Japanese population without diabetes into four glycometabolic categories. Each category had distinctly different insulin sensitivity and secretory characteristics [21]. However, prediabetes presents overlapping pathophysiology of impaired insulin sensitivity and secretion [25, 26]. Although screening tools for prediabetes have been developed [14, 16–20], this is the first study to develop a model to identify the glucose metabolism status of individuals without diabetes. This model encourages individuals to understand their glucose metabolism status and learn how they should change their lifestyle to prevent diabetes.

Second, the model requires only 10 questions about lifestyle and physical information that can be answered easily. Unlike diabetics who need to take their medications, nondiabetic people have no strong motivation or coercion to take screening tests. Clinical measurement values, such as fasting plasma glucose and abdominal circumference, are valid predictors of glucose metabolism status [14, 17–20]. However, the need to link a tool to clinical laboratory data may limit their scope of use. Moreover, in the questionnaire tool, variables that the user cannot remember may reduce the user's motivation. The simplicity of our tool helps individuals expand their opportunities to know their glucose metabolism status.

A systematic review of risk assessment tools for detecting prediabetes reported a mean AUC of 0.7 (ranging from 0.66 to 0.75) [27]. Meanwhile, in this study, the AUC for classifying category 1 (the best glucose metabolism group) and the other categories (impaired glucose metabolism groups) was 0.68 in the random forest model using 10 variables. In the external validation, the AUC was 0.66. However, due to the aforementioned differences from previous studies, this model has its own merits. In addition, some previous tools used the history of hyperglycemia and hypertension as variables [19, 28–31]. These have a clear association with diabetes risk and may contribute to improving the model performance. However, the population of this study excluded patients with hyperglycemic and hypertensive, which may also have affected the performance of the models. The AUC for classifying category 1 and the AUC for classifying category 4 were higher than those for classifying categories 1 and 4. Category 1 has the best glucose metabolism status, while category 4 has the worst glucose metabolism status with low insulin secretion and sensitivity [21]. Categories 2 and 3 are intermediate. Therefore, categories 1 and 4 may have been easier to identify.

Ten variables used in the random forest model are suggested to be associated with glucose metabolic status and diabetes risk. Variables included age, BMI, and height. Aging diminishes the ability to secrete insulin [11, 32], whereas obesity decreases insulin sensitivity [10, 33–35]. Cohort studies in Europe and Israel reported that height and risk of type 2 diabetes are inversely correlated [36, 37]. Sleep, diet, and lifestyle variables were also employed; laboratory interventions of circadian disruption were found to attenuate insulin sensitivity and insulin secretion [38–40]. Sleep duration is related to the risk of developing type 2 diabetes. Sleeping 7–8 hours per day has the lowest risk. [41]. Insomnia disorder with short sleep duration is associated with a higher risk of type 2 diabetes [42]. In addition, bedtime mobile phone use decreases sleep quality [43, 44]. Therefore, screen time at bedtime may be associated with glucose metabolism. However, the association has not been reported. This study is the first to suggest an association between glucose metabolic status and mobile phone and tablet computer use at bedtime. Preference for rice or bread is a question that reflects an individual's dietary styles. Rice is the primary source of carbohydrates for Asians. There is a positive association between rice intake and the development of type 2 diabetes [45]. Replacing refined grains with whole grains is recommended for diabetes prevention [46]. The relationship between diabetes and various dietary styles, such as the Mediterranean diet and vegetarian diet, has been studied [47, 48]. However, there is no single optimal dietary style [49, 50]. Further research is needed to determine the appropriate dietary style for each person. The frequency of soup intake may also be a factor related to dietary style. Tea is rich in polyphenols and caffeine. Several in vitro studies have shown that tea components enhance insulin sensitivity and insulin secretion [51–54]. Multiple epidemiological studies have shown that habitual consumption of tea decreases the risk of type 2 diabetes [55–58]. On the contrary, intervention trials have reported inconsistent results regarding the effects of tea on glucose metabolism [59–62]. Periodontal disease and oral inflammation worsen glycemic control and increase diabetes risk [63, 64]. Therefore, oral hygiene habits, such as frequency of toothbrush replacement, may be important in maintaining glucose metabolism status. However, their association has not been reported [65]. This study suggests for the first time that oral hygiene habits may be associated with glucose metabolism status. In this study, we developed models using a wide range of lifestyle and physical information, including unknown diabetes risk factors. From the aforementioned considerations, the 10 variables selected for the random forest model are reasonable. However, some lifestyles do not clearly present an association with glucose metabolism status. Further studies should clarify the causal relationship and molecular mechanism.

This study had some limitations. First, the study was aimed at Japanese people, and its application to other countries and ethnic groups is limited. In particular, the questions regarding diet correspond to Japanese food. Further research is needed to expand the scope of application. Second, the participants were recruited volunteers rather than randomly selected population-based samples. Third, the questionnaire was not validated, so measurement errors may have occurred. Nevertheless, the model was validated by external validation using data of people from another region of Japan. Therefore, the robustness of the model was confirmed. Notably, this model should be used for screening, and accurate diagnosis should be made by clinical tests, such as OGTT [13]. Despite the increasing number of patients with diabetes worldwide [1], impaired glucose metabolism is being overlooked because of its asymptomatic nature [66]. Understanding one's glucose metabolism status may provoke stronger behavioral motivation than vague lifestyle-related improvement suggestions.

## 5. Conclusions

In this study, we developed a model to identify glucose metabolism status of nondiabetics using a simple questionnaire. The model had the following two features. (i) It identified glucose metabolism status, i.e., insulin sensitivity and secretion, rather than prediabetes. (ii) It required only 10 factors, which were only questions about lifestyle and physical information that could be answered on the spot. These variables were selected using a random forest. The 10 factors were age, height, BMI, and the following questions: “Do you wake up in the middle of the night,” “Which do you usually eat: rice or bread,” “Frequency of tea intake per week at lunch,” “Do you wake up late on nonworking day,” “Frequency of mobile phone and tablet computer use at bedtime,” “Frequency of soup intake,” and “Frequency of toothbrush replacement.” Some factors do not have previously reported associations with glucose metabolism status. Thus, this study suggested new factors that can be associated with glucose metabolism status. The AUC for identifying categories with impaired glucose metabolism was 0.68. In the external validation, the AUC was 0.66, and the robustness of the model has been demonstrated. This model can be used by anyone, anywhere, by answering simple questions. This model provides an opportunity for many nondiabetic individuals to identify their glucose metabolism status. That can lead them to start improving their lifestyle to reduce their diabetes risk. The questionnaire of this study was aimed at Japanese people. In particular, the dietary questions were based on Japanese food. Therefore, the model's application to other countries and ethnic groups may be limited. Further research is needed to acquire and analyze data from different populations in order to expand the scope of application.

## Figures and Tables

**Table 1:** Review of the recent and important studies on prediabetes screening.

Ref. no.	Screening target	Factors	Models	Tool challenges
[14]	FPG 100–125 mg/dL, 120 mPG 100–125 mg/dL, or HbA1c 5.7–6.4%	25 of socioeconomic, clinical, and biochemical factors	RF, GBM, LR, and ANN	Invasive measurement factors were required for screening
[16]	FPG ≥100 mg/dL	Global diet quality score, age, smoking, alcohol drinking, unable to walk, use of rations card, time spent in sedentary activities	RF, GLMM, LASSO, and EN	Well-trained interviewers were needed to obtain dietary information
[17]	HbA1c 5.7–6.4%	Age, sex, BMI, waist circumference, and blood pressure	RF, GBM, XGB, LR, and DL	Lack of individuals with high blood glucose levels from screening targets Some of factors could not be answered on the spot and may require the linkage of the laboratory data
[18]	FPG 110–125 mg/dL or HbA1c 5.7–6.4%	Age, BMI, waist-to-hip ratio, systolic blood pressure, waist circumference, sleep duration, smoking status, and vigorous recreational activity time per week	XGB and LR	Lack of individuals with hyperglycemia after glucose loading from screening targets Some of factors could not be answered on the spot and may require the linkage of the laboratory data
[19]	FPG ≥110 mg/dL or 120 mPG ≥140 mg/dL	Age, sex, BMI, smoking, FPG, fasting plasma triglyceride level, and history of high FPG	LR	Research participants were limited to staffs in an oil field in China invasive measurement factors were required for screening
[20]	FPG 100–125 mg/dL, HbA1c 5.7–6.4%, or 120 mPG 140–199 mg/dL	Semiquantitative food frequency questionnaire answers and clinical and anthropometric measurements scores	LR	Well-trained interviewers were needed to obtain dietary information Invasive measurement factors were required for screening

Abbreviation: FPG: fasting plasma glucose level; 120 mPG: 120-min postload plasma glucose level during OGTT; HbA1c: hemoglobin A1c; BMI: body mass index; RF: random forest; GBM: gradient boosting machine; LR: logistic regression; ANN: artificial neural network; GLMM: generalized linear mixed model; LASSO: least absolute shrinkage and selection operator; EN: elastic net; XGB: XGBoost; DL: deep learning.

**Table 2:** Characteristics of the participants in each glycometabolic category.

	Category 1	Category 2	Category 3	Category 4
*n*	448	206	133	190
Sex (% women)	53.1	56.3	40.6	44.2
Age (years)	42.3 (41.2–43.4)	43.8 (42.3–45.3)	46.7 (44.7–48.6)^∗^	48.9 (47.4–50.4)^∗^
Height (m)	164.8 (164.1–165.6)	164.8 (163.6–166.0)	165.6 (164.2–166.9)	166.0 (164.9–167.1)
BMI (kg/m^2^)	21.4 (21.1–21.6)	23.5 (23.1–24.0)^∗^	21.6 (21.2–22.0)	23.4 (22.9–23.8)^∗^
30 mPG (mg/dL)	129.5 (127.9–131.1)	139.5 (137.7–141.2)^∗^	171.9 (169.7–174.2)^∗^	178.1 (175.5–180.8)^∗^
120 mPG (mg/dL)	94.4 (92.9–96.0)	127.6 (124.4–130.8)^∗^	99.3 (96.3–102.3)	134.6 (130.5–138.6)^∗^
Matsuda index	9.8 (9.4–10.2)	5.8 (5.4–6.3)^∗^	7.7 (7.3–8.1)^∗^	5.0 (4.6–5.4)^∗^

Data are presented as mean (95% confidence interval), percentage, or number of individuals. ^∗^*p* < 0.05 vs. category 1. Abbreviations: BMI: body mass index; x mPG: x-min postload plasma glucose level during the OGTT.

**Table 3:** Performances of the models for identifying glycometabolic category (95% confidence intervals).

Model	AUC for classifying category 1 and the others	AUC for classifying category 2 and the others	AUC for classifying category 3 and the others	AUC for classifying category 4 and the others	Mean of AUCs	Sensitivity to detect categories 2, 3, and 4	Specificity to detect category 1
Decision tree	0.63 (0.58-0.70)	0.68 (0.60-0.75)	0.56 (0.45-0.66)	0.61 (0.53-0.70)	0.62	0.71	0.41
Support vector machine	0.64 (0.57-0.70)	0.65 (0.57-0.73)	0.58 (0.47-0.68)	0.55 (0.48-0.64)	0.61	0.70	0.55
Random forest	0.69 (0.63-0.74)	0.68 (0.61-0.76)	0.63 (0.55-0.72)	0.67 (0.59-0.74)	0.67	0.70	0.46
XGBoost	0.62 (0.56-0.68)	0.58 (0.50-0.66)	0.59 (0.49-0.69)	0.60 (0.52-0.68)	0.60	0.70	0.45

**Table 4:** Performance of the random forest model using the ten variables (95% confidence interval).

Model	AUC for classifying category 1 and the others	AUC for classifying category 2 and the others	AUC for classifying category 3 and the others	AUC for classifying category 4 and the others	Mean of AUCs	Sensitivity to detect categories 2, 3, and 4	Specificity to detect category 1
Random forest using 10 variables	0.68 (0.62–0.75)	0.66 (0.58–0.73)	0.61 (0.51–0.70)	0.70 (0.62–0.77)	0.66	0.70	0.41

**Table 5:** Ten most important variables of the model and their importances.

Variable	Mean decrease in Gini coefficient
Body mass index	10.3
Age	8.1
Height	3.3
Do you wake up in the middle of the night?	3.1
Which do you usually eat: rice or bread?	2.5
Frequency of tea intake per week at lunch	2.1
Do you wake up late on nonworking day?	1.9
Frequency of mobile phone and tablet computer use at bedtime	1.4
Frequency of soup intake	1.4
Frequency of toothbrush replacement	0.8

**Table 6:** Performance of the random forest model using the ten variables in the external validation (95% confidence interval).

Model	AUC for classifying category 1 and the others	AUC for classifying category 2 and the others	AUC for classifying category 3 and the others	AUC for classifying category 4 and the others	Mean of AUCs	Sensitivity to detect categories 2, 3, and 4	Specificity to detect category 1
Random forest using 10 variables	0.66 (0.61–0.71)	0.57 (0.51–0.62)	0.60 (0.50–0.69)	0.64 (0.57–0.71)	0.62	0.70	0.55

## Data Availability

The data of participant characteristics and questionnaire used to support the findings of this study are currently under embargo, while the research findings are commercialized. Requests for data 12 months after publication of this article will be considered by the corresponding author.

## Supplementary Materials

Supplementary Materials Supplementary Figure 1: questionnaire on lifestyle and physical characteristics. Supplementary Table 1: characteristics of the preprocessed questionnaire answers for each glycometabolic category. Supplementary Table 2: characteristics of the participants in each glycometabolic category in the external validation. Data are presented as mean (95% confidence interval), percentage, or number of individuals. ^∗^*p* < 0.05 vs. category 1. Abbreviations: BMI: body mass index; x mPG: x-min postload plasma glucose level during the OGTT. Supplementary Table 3: characteristics of the questionnaire answers for each glycometabolic category used in the external validation of the random forest model.
