# Supplementary material for: Identifying Glucose Metabolism Status in Nondiabetic Japanese Adults Using Machine Learning Model with Simple Questionnaire
Source: Comput Math Methods Med. 2022 Sep 9;2022:1026121. doi: 10.1155/2022/1026121 (PMC9481387; doi:10.1155/2022/1026121)
Supplement: Supplementary Materials — Supplementary Figure 1: questionnaire on lifestyle and physical characteristics. Supplementary Table 1: characteristics of the preprocessed questionnaire answers for each glycometabolic category. Supplementary Table 2: characteristics of the participants in each glycometabolic category in the external validation. Data are presented as mean (95% confidence interval), percentage, or number of individuals. ∗p < 0.05 vs. category 1. Abbreviations: BMI: body mass index; x mPG: x-min postload plasma glucose level during the OGTT. Supplementary Table 3: characteristics of the questionnaire answers for each glycometabolic category used in the external validation of the random forest model. [file 1026121.f1.zip › STROBE-checklist_revised.docx]

STROBE Statement—checklist of items that should be included in reports of observational studies

|  | Item No. | Recommendation | Page  No. | Relevant text from manuscript |
| --- | --- | --- | --- | --- |
| **Title and abstract** | 1 | (*a*) Indicate the study’s design with a commonly used term in the title or the abstract | 1 | In this cross-sectional study, Japanese adults (aged 20–64 years) from Tokyo and surrounding areas were recruited. |
|  |  | (*b*) Provide in the abstract an informative and balanced summary of what was done and what was found | 1 | [abstract] |
| Introduction | | | |  |
| Background/rationale | 2 | Explain the scientific background and rationale for the investigation being reported | 2, 3 | In Introduction, we added a review of the recent and important studies on pre-diabetes screening tools and included a table to list the information (page 2 lines 55–page 3 lines 101). |
| Objectives | 3 | State specific objectives, including any prespecified hypotheses | 3 | In this study, we aimed to develop a machine learning model to identify glucose metabolism status in non-diabetic adults. The present study has two unique contributions. First, the factors of the model include only lifestyle and physical information that can be answered on the spot. Because invasive measurement factors or several factors are not needed, it can be easily and widely used by general population. Second, we identified glucose metabolism status rather than pre-diabetes. No tools have been reported to determine glucose metabolism status in non-diabetic individuals. |
| Methods | | | |  |
| Study design | 4 | Present key elements of study design early in the paper | 4 | In this cross-sectional study, we recruited Japanese adults without diabetes aged 20–64 years in Tokyo and the surrounding area in 2019. |
| Setting | 5 | Describe the setting, locations, and relevant dates, including periods of recruitment, exposure, follow-up, and data collection | 4 | In this cross-sectional study, we recruited Japanese adults without diabetes aged 20–64 years in Tokyo and the surrounding area in 2019.  Participants underwent height and weight measurements and 75 g OGTT. Blood sampling in the OGTT was performed before glucose loading and 30, 60, 90, and 120 minutes after glucose loading. Participants also completed a questionnaire on lifestyle and physical characteristics.  For external verification data, we recruited Japanese adults without diabetes aged 20–64 years in Hokkaido, Japan in 2021. The selection and exclusion criteria were the same. The same examinations and questionnaire were conducted on them. |
| Participants | 6 | (*a*) *Cohort study*—Give the eligibility criteria, and the sources and methods of selection of participants. Describe methods of follow-up  *Case-control study*—Give the eligibility criteria, and the sources and methods of case ascertainment and control selection. Give the rationale for the choice of cases and controls  *Cross-sectional study*—Give the eligibility criteria, and the sources and methods of selection of participants | 4 | Those with cardiovascular disorders, liver disorders, and kidney disorders, those taking medication, pregnant women, and lactating women were excluded. Diabetes was defined as a fasting plasma glucose level ≥ 126 mg/dL, 120 mPG ≥ 200 mg/dL, and/or the use of anti-diabetic medications [13]. |
|  |  | (*b*) *Cohort study*—For matched studies, give matching criteria and number of exposed and unexposed  *Case-control study*—For matched studies, give matching criteria and the number of controls per case | - | - |
| Variables | 7 | Clearly define all outcomes, exposures, predictors, potential confounders, and effect modifiers. Give diagnostic criteria, if applicable | 4, 5 | Participants were classified into the four glycometabolic categories based on plasma glucose concentrations and Matsuda index during the OGTT. The four categories were the objective variables of the models in this study. The classification criteria were as follows: condition A – 30 mPG <157 mg / dL and condition B – 120 mPG <126 mg / dL and Matsuda index> 4.97, category 1 satisfies conditions A and B, category 2 satisfies condition A but not condition B, category 3 satisfies condition B but not condition A, and category 4 satisfies neither condition A nor condition B.  For the explanatory variables, we used a dataset that included questionnaire responses, age, gender, height, and body mass index (BMI). The questionnaire consisted of 309 questions that did not require clinical examination data and could be answered easily on the spot (Supplementary figure 1). |
| Data sources/ measurement | 8* | For each variable of interest, give sources of data and details of methods of assessment (measurement). Describe comparability of assessment methods if there is more than one group | 4 | we recruited Japanese adults without diabetes aged 20–64 years in Tokyo and the surrounding area in 2019.  For external verification data, we recruited Japanese adults without diabetes aged 20–64 years in Hokkaido, Japan in 2021. |
| Bias | 9 | Describe any efforts to address potential sources of bias | 5 | The model performances were assessed using the testing dataset and verified using the external verification dataset. |
| Study size | 10 | Explain how the study size was arrived at | - | - |

Continued on next page

| Quantitative variables | 11 | Explain how quantitative variables were handled in the analyses. If applicable, describe which groupings were chosen and why | - | - |
| --- | --- | --- | --- | --- |
| Statistical methods | 12 | (*a*) Describe all statistical methods, including those used to control for confounding | 6 | The characteristics of each glycometabolic category and the OGTT values were compared using analysis of variance (ANOVA) with Dunnett’s test for multiple comparisons [24]. For the insulinogenic index and disposition index, outliers were excluded by Smirnov–Grubbs test. Spearman’s correlation test was used to calculate the relationships between the variables. A *p* value < 0.05 was considered to indicate statistical significance. |
|  |  | (*b*) Describe any methods used to examine subgroups and interactions | - | - |
|  |  | (*c*) Explain how missing data were addressed | 5 | Those who did not answer the questionnaire and those who answered less than 90% of the questionnaire were excluded from the analysis.  As a pretreatment for the analysis, missing answers were replaced with the mode. |
|  |  | (*d*) *Cohort study*—If applicable, explain how loss to follow-up was addressed  *Case-control study*—If applicable, explain how matching of cases and controls was addressed  *Cross-sectional study*—If applicable, describe analytical methods taking account of sampling strategy | - | - |
|  |  | (*e*) Describe any sensitivity analyses | - | - |
| Results | | | | |
| Participants | 13* | (a) Report numbers of individuals at each stage of study—eg numbers potentially eligible, examined for eligibility, confirmed eligible, included in the study, completing follow-up, and analysed | 6, 7 | Of the total of 977 eligible participants in the original dataset, the glycometabolic categories 1, 2, 3, and 4 accounted for 46% (n = 448), 21% (n = 206), 14% (n = 133), and 19% (n = 190), respectively (Table 2). |
|  |  | (b) Give reasons for non-participation at each stage | - | - |
|  |  | (c) Consider use of a flow diagram | - | - |
| Descriptive data | 14* | (a) Give characteristics of study participants (eg demographic, clinical, social) and information on exposures and potential confounders | 6, 7 | Of the total of 977 eligible participants in the original dataset, the glycometabolic categories 1, 2, 3, and 4 accounted for 46% (n = 448), 21% (n = 206), 14% (n = 133), and 19% (n = 190), respectively (Table 2). Regarding the age, categories 3 and 4 were significantly higher than category 1. Regarding the BMI, categories 2 and 4 were significantly higher than category 1. The questionnaire answers were obtained from 977 participants. None of the subjects had more than 1% of missing answers. Missing answers were replaced with the mode. Nominal variable answers were split and converted to dummy variables. If two variables had a correlation coefficient greater than 0.7, one of them was excluded. Supplementary Table 1 shows the characteristics of the preprocessed 279 questionnaire answers in each category. |
|  |  | (b) Indicate number of participants with missing data for each variable of interest | - | - |
|  |  | (c) *Cohort study*—Summarise follow-up time (eg, average and total amount) | - | - |
| Outcome data | 15* | *Cohort study*—Report numbers of outcome events or summary measures over time | - | - |
|  |  | *Case-control study—*Report numbers in each exposure category, or summary measures of exposure | - | - |
|  |  | *Cross-sectional study—*Report numbers of outcome events or summary measures | 7 | Table 1. Characteristics of the participants in each glycometabolic category |
| Main results | 16 | (*a*) Give unadjusted estimates and, if applicable, confounder-adjusted estimates and their precision (eg, 95% confidence interval). Make clear which confounders were adjusted for and why they were included | 8 | “Model performance using 10 variables”  Its AUCs (95% confidence intervals) to classify category 1 and others, category 2 and others, category 3 and others, and category 4 and others were 0.68 (0.62–0.75), 0.66 (0.58–0.73), 0.61 (0.51–0.70), and 0.70 (0.62–0.77), respectively. |
|  |  | (*b*) Report category boundaries when continuous variables were categorized | - | - |
|  |  | (*c*) If relevant, consider translating estimates of relative risk into absolute risk for a meaningful time period | - | - |

Continued on next page

| Other analyses | 17 | Report other analyses done—eg analyses of subgroups and interactions, and sensitivity analyses | 9 | “Model performance in the external validation”  Its AUCs (95% confidence intervals) to classify category 1 and others, category 2 and others, category 3 and others, and category 4 and others were 0.66 (0.61–0.71), 0.57 (0.51–0.62), 0.60 (0.50–0.69), 0.64 (0.57–0.71) (Table 6). |
| --- | --- | --- | --- | --- |
| Discussion | | | | |
| Key results | 18 | Summarise key results with reference to study objectives | 10 | In this study, we identified the glucose metabolism status of non-diabetic Japanese adults using a questionnaire. This model had two unique features. The first is it identifies the glucose metabolism status of individuals without diabetes. Second, the model requires only 10 questions about lifestyle and physical information that can be answered easily. |
| Limitations | 19 | Discuss limitations of the study, taking into account sources of potential bias or imprecision. Discuss both direction and magnitude of any potential bias | 10, 11 | This study had some limitations. First, the study was aimed at Japanese people, and its application to other countries and ethnic groups is limited. In particular, the questions regarding diet correspond to Japanese food. Further research is needed to expand the scope of application. Second, the participants were recruited volunteers rather than randomly selected population-based samples. Third, the questionnaire was not validated, so measurement errors may have occurred. Nevertheless, the model was validated by external validation using data of people from another region of Japan. Therefore, the robustness of the model was confirmed. |
| Interpretation | 20 | Give a cautious overall interpretation of results considering objectives, limitations, multiplicity of analyses, results from similar studies, and other relevant evidence | 10 | Although screening tools for pre-diabetes have been developed [14–19], this is the first study to develop a model to identify the glucose metabolism status of individuals without diabetes. This model encourages individuals to understand their glucose metabolism status and learn how they should change their lifestyle to prevent diabetes. |
| Generalisability | 21 | Discuss the generalisability (external validity) of the study results | 10, 11 | First, the study was aimed at Japanese people, and its application to other countries and ethnic groups is limited. In particular, the questions regarding diet correspond to Japanese food. Further research is needed to expand the scope of application. Second, the participants were recruited volunteers rather than randomly selected population-based samples. Third, the questionnaire was not validated, so measurement errors may have occurred. Nevertheless, the model was validated by external validation using data of people from another region of Japan. Therefore, the robustness of the model was confirmed. |
| Other information | |  | | |
| Funding | 22 | Give the source of funding and the role of the funders for the present study and, if applicable, for the original study on which the present article is based | 12 | This study was funded by Suntory Global Innovation Center Limited. |

*Give information separately for cases and controls in case-control studies and, if applicable, for exposed and unexposed groups in cohort and cross-sectional studies.

**Note:** An Explanation and Elaboration article discusses each checklist item and gives methodological background and published examples of transparent reporting. The STROBE checklist is best used in conjunction with this article (freely available on the Web sites of PLoS Medicine at http://www.plosmedicine.org/, Annals of Internal Medicine at http://www.annals.org/, and Epidemiology at http://www.epidem.com/). Information on the STROBE Initiative is available at www.strobe-statement.org.
