# Supplementary material for: Identifying Glucose Metabolism Status in Nondiabetic Japanese Adults Using Machine Learning Model with Simple Questionnaire
Source: Comput Math Methods Med. 2022 Sep 9;2022:1026121. doi: 10.1155/2022/1026121 (PMC9481387; doi:10.1155/2022/1026121)
Supplement: Supplementary Materials — Supplementary Figure 1: questionnaire on lifestyle and physical characteristics. Supplementary Table 1: characteristics of the preprocessed questionnaire answers for each glycometabolic category. Supplementary Table 2: characteristics of the participants in each glycometabolic category in the external validation. Data are presented as mean (95% confidence interval), percentage, or number of individuals. ∗p < 0.05 vs. category 1. Abbreviations: BMI: body mass index; x mPG: x-min postload plasma glucose level during the OGTT. Supplementary Table 3: characteristics of the questionnaire answers for each glycometabolic category used in the external validation of the random forest model. [file 1026121.f1.zip › Supplementary_materials_revised.docx]

**Supplementary Figure 1. Questionnaire on lifestyle and physical characteristics**

Please answer the following questions about your lifestyle and physical condition over the last month.

Please provide only one option per question except for the last question in this survey, for which you can provide multiple answers.

[Exercise habits]

◆ Activity intensity

| How did you spend your working days? | 1. Mainly static activities (Most of the work was done sitting down. There was little movement, or it was a static way to move while sitting down, such as driving). | 2. Exercise moderately (You spent a lot of time sitting, but you had the opportunity to exercise for commuting, moving in the workplace, standing work / customer service, shopping, housework, etc.) | 3. Actively moving (You spent a lot of time moving and standing for work and housework. You spent a lot of time on farm work, carrying heavy luggage, going up and down stairs, etc.) |
| --- | --- | --- | --- |
| How did you spend your non-working days? | 1. Mainly static activities (Most of the time was spent sitting, and most of the activities were static, such as watching TV, reading, and driving). | 2. Light exercise and walking | 3. Active engagements such as mountain climbing, running, swimming, tennis, and soccer. |

◆ Travel method and time required (working days)

| On foot | 1. 90 min or more | 2. 60-89 min | 3. 30-59 min | 4. 5 – 29 min | 5. Less than 5 min |
| --- | --- | --- | --- | --- | --- |
| Running | 1. 90 min or more | 2. 60-89 min | 3. 30-59 min | 4. 5 – 29 min | 5. Less than 5 min |
| By bicycle | 1. 90 min or more | 2. 60-89 min | 3. 30-59 min | 4. 5 – 29 min | 5. Less than 5 min |
| By train / bus | 1. 90 min or more | 2. 60-89 min | 3. 30-59 min | 4. 5 – 29 min | 5. Less than 5 min |
| By car / motorcycle | 1. 90 min or more | 2. 60-89 min | 3. 30-59 min | 4. 5 – 29 min | 5. Less than 5 min |

◆ Travel method and time required (non-working days)

| On foot | 1. 90 min or more | 2. 60-89 min | 3. 30-59 min | 4. 5 – 29 min | 5. Less than 5 min |
| --- | --- | --- | --- | --- | --- |
| Running | 1. 90 min or more | 2. 60-89 min | 3. 30-59 min | 4. 5 – 29 min | 5. Less than 5 min |
| By bicycle | 1. 90 min or more | 2. 60-89 min | 3. 30-59 min | 4. 5 – 29 min | 5. Less than 5 min |
| By train / bus | 1. 90 min or more | 2. 60-89 min | 3. 30-59 min | 4. 5 – 29 min | 5. Less than 5 min |
| By car / motorcycle | 1. 90 min or more | 2. 60-89 min | 3. 30-59 min | 4. 5 – 29 min | 5. Less than 5 min |

◆ Duration of exercise (weekly total)

| Walking | 1. 90 min or more | 2. 60-89 min | 3. 30-59 min | 4. 5 – 29 min | 5. Less than 5 min |
| --- | --- | --- | --- | --- | --- |
| Running | 1. 90 min or more | 2. 60-89 min | 3. 30-59 min | 4. 5 – 29 min | 5. Less than 5 min |
| Swimming | 1. 90 min or more | 2. 60-89 min | 3. 30-59 min | 4. 5 – 29 min | 5. Less than 5 min |
| Strength training | 1. 90 min or more | 2. 60-89 min | 3. 30-59 min | 4. 5 – 29 min | 5. Less than 5 min |
| Other exercise | 1. 90 min or more | 2. 60-89 min | 3. 30-59 min | 4. 5 – 29 min | 5. Less than 5 min |

◆ Please select the one that applies to your exercise situation and awareness of exercise.

| You joined a sports gym | 1. Yes | 2. No |  |  |  |
| --- | --- | --- | --- | --- | --- |
| You own athletic shoes or gym clothes | 1. Yes | 2. No |  |  |  |
| Time of exercise | 1. before meals | 2. after meals | 3. both before and after meals | 4. not decided in particular | 5. No exercise habits |

[Sleep habits and drowsiness]

◆ Sleep habits

| Average sleeping time | 1. 8 hours or more | 2. 7 hours to less than 8 hours | 3. 6 hours to less than 7 hours | 4. 5 hours to less than 6 hours | 5. less than 5 hours |
| --- | --- | --- | --- | --- | --- |
| Variation in bedtime | 1. almost the same time every day. | 2. different time on working days and non-working days | 3. Different time each day |  |  |
| Falling asleep | 1. You don't have trouble falling asleep | 2. You have trouble falling asleep |  |  |  |
| Variation in sleep duration | 1. almost the same duration every day | 2. Different duration on working days and non-working days | 3. Different duration each day |  |  |

◆ Sleep habits and drowsiness

| Feel that you get enough sleep | 1. Always | 2. Frequently | 3. Sometimes | 4. Seldom | 5. Never |
| --- | --- | --- | --- | --- | --- |
| Stay up late | 1. Always | 2. Frequently | 3. Sometimes | 4. Seldom | 5. Never |
| Take sleeping pills | 1. Always | 2. Frequently | 3. Sometimes | 4. Seldom | 5. Never |
| Drink alcohol to sleep | 1. Always | 2. Frequently | 3. Sometimes | 4. Seldom | 5. Never |
| You fall asleep and wake up right away | 1. Always | 2. Frequently | 3. Sometimes | 4. Seldom | 5. Never |
| Frequency of mobile phone and tablet computer use at bedtime | 1. Always | 2. Frequently | 3. Sometimes | 4. Seldom | 5. Never |
| Spend in a bright room until just before going to bed | 1. Always | 2. Frequently | 3. Sometimes | 4. Seldom | 5. Never |
| Wake up in the middle of the night | 1. Always | 2. Frequently | 3. Sometimes | 4. Seldom | 5. Never |
| Wake up during the night because you feel the need to go to the toilet. | 1. Always | 2. Frequently | 3. Sometimes | 4. Seldom | 5. Never |
| Wake up in the middle of the night and have difficulty sleeping afterwards | 1. Always | 2. Frequently | 3. Sometimes | 4. Seldom | 5. Never |
| Feel suffocation during sleep | 1. Always | 2. Frequently | 3. Sometimes | 4. Seldom | 5. Never |
| When you wake up in the morning still feel sleepy and tired | 1. Always | 2. Frequently | 3. Sometimes | 4. Seldom | 5. Never |
| Wake up refreshed in the morning | 1. Always | 2. Frequently | 3. Sometimes | 4. Seldom | 5. Never |
| Feel sticky in the mouth when you wake up in the morning | 1. Always | 2. Frequently | 3. Sometimes | 4. Seldom | 5. Never |
| Not hungry when you wake up in the morning. | 1. Always | 2. Frequently | 3. Sometimes | 4. Seldom | 5. Never |
| Become drowsy after a meal | 1. Always | 2. Frequently | 3. Sometimes | 4. Seldom | 5. Never |
| Long-lasting drowsiness after a meal | 1. Always | 2. Frequently | 3. Sometimes | 4. Seldom | 5. Never |
| Feel too sleepy to stay awake, even while working or doing household chores | 1. Always | 2. Frequently | 3. Sometimes | 4. Seldom | 5. Never |
| Wake up late on non-working day | 1. Always | 2. Frequently | 3. Sometimes | 4. Seldom | 5. Never |
| You cannot wake up on non-working day without an alarm clock. | 1. Always | 2. Frequently | 3. Sometimes | 4. Seldom | 5. Never |
| Take a nap on non-working day | 1. Always | 2. Frequently | 3. Sometimes | 4. Seldom | 5. Never |
| Have habits to sleep well | 1. Yes | 2. No |  |  |  |
| Have been told that you have stopped breathing during sleep | 1. Yes | 2. No |  |  |  |
| Have been told you snores | 1. Yes | 2. No |  |  |  |
| Have been told you are bruxing your teeth during sleeping | 1. Yes | 2. No |  |  |  |

[Dietary habits]

◆ Frequency and duration

| Frequency of eating breakfast per week | 1. Every day | 2. 4-6 times a week | 3. 2-3 times a week | 4. Once a week | 5. Less than once a week |
| --- | --- | --- | --- | --- | --- |
| Time required for breakfast | 1. 30 min or more | 2. 20 min to less than 30 min | 3. 10 min to less than 20 min | 4. 5 min to less than 10 min | 5. less than 5 min |
| Frequency of eating lunch per week | 1. Every day | 2. 4-6 times a week | 3. 2-3 times a week | 4. Once a week | 5. Less than once a week |
| Time required for lunch | 1. 30 min or more | 2. 20 min to less than 30 min | 3. 10 min to less than 20 min | 4. 5 min to less than 10 min | 5. less than 5 min |
| Frequency of eating supper per week | 1. Every day | 2. 4-6 times a week | 3. 2-3 times a week | 4. Once a week | 5. Less than once a week |
| Time required for supper | 1. 30 min or more | 2. 20 min to less than 30 min | 3. 10 min to less than 20 min | 4. 5 min to less than 10 min | 5. less than 5 min |
| Frequency of eating after-supper snacks per week | 1. Every day | 2. 4-6 times a week | 3. 2-3 times a week | 4. Once a week | 5. Less than once a week |
| Time required for after-supper snacks | 1. 30 min or more | 2. 20 min to less than 30 min | 3. 10 min to less than 20 min | 4. 5 min to less than 10 min | 5. less than 5 min |
| Frequency of snacking per week (between breakfast and lunch or between lunch and dinner) | 1. Every day | 2. 4-6 times a week | 3. 2-3 times a week | 4. Once a week | 5. Less than once a week |
| Time required for snacks (between breakfast and lunch or between lunch and dinner) | 1. 30 min or more | 2. 20 min to less than 30 min | 3. 10 min to less than 20 min | 4. 5 min to less than 10 min | 5. less than 5 min |

◆ Meal timing

| Eat breakfast at about the same time | 1. Yes | 2. No | 3. You eat breakfast less than once a week |
| --- | --- | --- | --- |
| Eat lunch at about the same time | 1. Yes | 2. No | 3. You eat lunch less than once a week |
| Eat supper at about the same time | 1. Yes | 2. No | 3. You eat supper less than once a week |
| Eat after-supper snacks at about the same time | 1. Yes | 2. No | 3. You eat after-supper snacks less than once a week |
| Eat snacks (between breakfast and lunch or between lunch and dinner) at about the same time | 1. Yes | 2. No | 3. You eat snacks less than once a week |

◆ Dietary habits

| Frequency of consuming yogurt and probiotics drinks | 1. Every day | 2. 4-6 times a week | 3. 2-3 times a week | 4. once a week | 5. less than once a week |
| --- | --- | --- | --- | --- | --- |
| Which do you usually eat: rice or bread? | 1. usually eat rice | 2. eat rice more often than bread | 3. about the same frequency | 4 eat bread more often than rice | 5. usually eat bread |
| Amount of rice you usually ate compared to one serving of rice at a restaurant | 1. more | 2. somewhat more | 3. about the same | 4. somewhat less | 5. less |
| Amount of main dish you usually ate compared to one serving of main dish at a restaurant | 1. more | 2. somewhat more | 3. about the same | 4. somewhat less | 5. less |
| Frequency of soup intake | 1. more | 2. somewhat more | 3. about the same | 4. somewhat less | 5. less |
| Frequency of noodles intake | 1. more | 2. somewhat more | 3. about the same | 4. somewhat less | 5. less |
| Frequency of vegetable intake | 1. more | 2. somewhat more | 3. about the same | 4. somewhat less | 5. less |
| Amount of soup left in noodle dish | 1. very little left | 2. 20-30% | 3. About half | 4. 70-80% | 5. almost all left |

◆ Characteristics of your diet

| Try to eat well-balanced diet | 1. Yes | 2. No |
| --- | --- | --- |
| Try not to overeat | 1. Yes | 2. No |
| Try to choose low-carbohydrate diet | 1. Yes | 2. No |
| faster to eat than others | 1. Yes | 2. No |
| Try to choose low-salt diet | 1. Yes | 2. No |
| Try to eat vegetables first | 1. Yes | 2. No |
| Often eat sweets | 1. Yes | 2. No |
| Often eat seafood, tofu, and beans. | 1. Yes | 2. No |
| Eat more than other people | 1. Yes | 2. No |
| Often have a large serving and extra serving | 1. Yes | 2. No |
| Feel unsatisfied without rice dish or noodles after drinking alcohol | 1. Yes | 2. No |
| Finish supper two hours before going to bed | 1. Yes | 2. No |
| Meals served one by one | 1. Yes | 2. No |
| Often eat after-meal dessert | 1. Yes | 2. No |
| Often add salt or soy sauce to dishes. | 1. Yes | 2. No |
| Try to choose meat with less fat and remove excess fat. | 1. Yes | 2. No |
| Often eat until you're full. | 1. Yes | 2. No |
| Often eat rice dish or noodle or sweets after drinking alcohol. | 1. Yes | 2. No |
| Hardly ever cook at home | 1. Yes | 2. No |
| Try to chew well and eat | 1. Yes | 2. No |
| After supper, go to bed on a full stomach. | 1. Yes | 2. No |
| Try to eat a lot of vegetables | 1. Yes | 2. No |

◆ Meal details

|  | Days of intake per week | | | | | |
| --- | --- | --- | --- | --- | --- | --- |
|  | Don’t eat | More than one day a month but less than one day a week | One day a week | 2 to 3 days a week | 4 to 6 days a week | Almost every day a week |
| ■ Rice | | | | | | |
| Rice ball | 0 | 1 | 2 | 3 | 4 | 5 |
| Rice porridge and risotto | 0 | 1 | 2 | 3 | 4 | 5 |
| Japanese seasoned rice with vegetables | 0 | 1 | 2 | 3 | 4 | 5 |
| Rice (white rice, brown rice, barley rice, etc.) | 0 | 1 | 2 | 3 | 4 | 5 |
| Fried rice, pilaf, and omelet rice | 0 | 1 | 2 | 3 | 4 | 5 |
| Sushi | 0 | 1 | 2 | 3 | 4 | 5 |
| Bowl of rice with topping | 0 | 1 | 2 | 3 | 4 | 5 |
| Curry rice, hashed beef rice | 0 | 1 | 2 | 3 | 4 | 5 |
| ■ Bread | | | | | | |
| Plain bread | 0 | 1 | 2 | 3 | 4 | 5 |
| Bread rolls, croissants, French bread | 0 | 1 | 2 | 3 | 4 | 5 |
| Sweet bread, side dish bread, sandwiches, hamburgers, and pizza. | 0 | 1 | 2 | 3 | 4 | 5 |
| ■ Noodles, etc. | | | | | | |
| Noodles (ramen, udon, soba, pasta, etc.) | 0 | 1 | 2 | 3 | 4 | 5 |
| Okonomiyaki (savory Japanese-style of pancake.) | 0 | 1 | 2 | 3 | 4 | 5 |
| Cereal | 0 | 1 | 2 | 3 | 4 | 5 |
| ■ Soup | | | | | | |
| Soup | 0 | 1 | 2 | 3 | 4 | 5 |
| Stew | 0 | 1 | 2 | 3 | 4 | 5 |
| Japanese hot pot dish | 0 | 1 | 2 | 3 | 4 | 5 |
| ■ Main and side dishes | | | | | | |
| Simmered Food (main dishes: pot-au-feu, meat potato, etc.) | 0 | 1 | 2 | 3 | 4 | 5 |
| Simmered Food (side dishes: hijiki seaweed, dried strips of radish, etc.) | 0 | 1 | 2 | 3 | 4 | 5 |
| Deep-fried food | 0 | 1 | 2 | 3 | 4 | 5 |
| Grilled fish (grilled fish, grilled meat, hamburger steak, fried egg, etc.) | 0 | 1 | 2 | 3 | 4 | 5 |
| Stir-fried food (stir-fried vegetables, mapo tofu, roasted eggs, etc.) | 0 | 1 | 2 | 3 | 4 | 5 |
| Boiled and steamed food (hot vegetables, boiled eggs, chawanmushi, shumai, etc.) | 0 | 1 | 2 | 3 | 4 | 5 |
| Raw food (sashimi, natto, tofu, cod roe, etc.) | 0 | 1 | 2 | 3 | 4 | 5 |
| Salad, raw vegetables | 0 | 1 | 2 | 3 | 4 | 5 |
| Vinegared food | 0 | 1 | 2 | 3 | 4 | 5 |
| Ohitashi (boiled spinach seasoned with soy sauce) | 0 | 1 | 2 | 3 | 4 | 5 |
| Japanese pickles | 0 | 1 | 2 | 3 | 4 | 5 |
| ■ Cheese, fruits, desserts, sweets, etc. | | | | | | |
| Cheese, dried fruits, nuts | 0 | 1 | 2 | 3 | 4 | 5 |
| Fruits (except berries) | 0 | 1 | 2 | 3 | 4 | 5 |
| Fruits (berries) | 0 | 1 | 2 | 3 | 4 | 5 |
| Yogurt, pudding, jelly, ice cream | 0 | 1 | 2 | 3 | 4 | 5 |
| Cakes, pies, tarts | 0 | 1 | 2 | 3 | 4 | 5 |
| Traditional Japanese sweets | 0 | 1 | 2 | 3 | 4 | 5 |
| Fried confectionery | 0 | 1 | 2 | 3 | 4 | 5 |

[Water intake]

◆ Drink intake

| Time | Beverage | frequency | | | | | | amount | | | | | |
| --- | --- | --- | --- | --- | --- | --- | --- | --- | --- | --- | --- | --- | --- |
|  |  | Don’t drink | More than one day a month but less than one day a week | One day a week | 2 to 3 days a week | 4 to 6 days a week | Almost every day a week | Don’t drink | about 100 mL | about 200 mL | About 350 mL | 500 mL or more |  |
| 1. After waking up-before breakfast | Tea | 0 | 1 | 2 | 3 | 4 | 5 | A | B | C | D | E |  |
|  | Water | 0 | 1 | 2 | 3 | 4 | 5 | A | B | C | D | E |  |
|  | Coffee | 0 | 1 | 2 | 3 | 4 | 5 | A | B | C | D | E |  |
|  | Milk and yogurt drink | 0 | 1 | 2 | 3 | 4 | 5 | A | B | C | D | E |  |
|  | Fruit and vegetable drinks | 0 | 1 | 2 | 3 | 4 | 5 | A | B | C | D | E |  |
|  | Other soft drinks | 0 | 1 | 2 | 3 | 4 | 5 | A | B | C | D | E |  |
| 2. At breakfast | Tea | 0 | 1 | 2 | 3 | 4 | 5 | A | B | C | D | E |  |
|  | Water | 0 | 1 | 2 | 3 | 4 | 5 | A | B | C | D | E |  |
|  | Coffee | 0 | 1 | 2 | 3 | 4 | 5 | A | B | C | D | E |  |
|  | Milk and yogurt drink | 0 | 1 | 2 | 3 | 4 | 5 | A | B | C | D | E |  |
|  | Fruit and vegetable drinks | 0 | 1 | 2 | 3 | 4 | 5 | A | B | C | D | E |  |
|  | Other soft drinks | 0 | 1 | 2 | 3 | 4 | 5 | A | B | C | D | E |  |
| 3. After breakfast-before lunch | Tea | 0 | 1 | 2 | 3 | 4 | 5 | A | B | C | D | E |  |
|  | Water | 0 | 1 | 2 | 3 | 4 | 5 | A | B | C | D | E |  |
|  | Coffee | 0 | 1 | 2 | 3 | 4 | 5 | A | B | C | D | E |  |
|  | Milk and yogurt drink | 0 | 1 | 2 | 3 | 4 | 5 | A | B | C | D | E |  |
|  | Fruit and vegetable drinks | 0 | 1 | 2 | 3 | 4 | 5 | A | B | C | D | E |  |
|  | Other soft drinks | 0 | 1 | 2 | 3 | 4 | 5 | A | B | C | D | E |  |
| 4. At lunch | Tea | 0 | 1 | 2 | 3 | 4 | 5 | A | B | C | D | E |  |
|  | Water | 0 | 1 | 2 | 3 | 4 | 5 | A | B | C | D | E |  |
|  | Coffee | 0 | 1 | 2 | 3 | 4 | 5 | A | B | C | D | E |  |
|  | Milk and yogurt drink | 0 | 1 | 2 | 3 | 4 | 5 | A | B | C | D | E |  |
|  | Fruit and vegetable drinks | 0 | 1 | 2 | 3 | 4 | 5 | A | B | C | D | E |  |
|  | Other soft drinks | 0 | 1 | 2 | 3 | 4 | 5 | A | B | C | D | E |  |
| 5.　After lunch-before dinner | Tea | 0 | 1 | 2 | 3 | 4 | 5 | A | B | C | D | E |  |
|  | Water | 0 | 1 | 2 | 3 | 4 | 5 | A | B | C | D | E |  |
|  | Coffee | 0 | 1 | 2 | 3 | 4 | 5 | A | B | C | D | E |  |
|  | Milk and yogurt drink | 0 | 1 | 2 | 3 | 4 | 5 | A | B | C | D | E |  |
|  | Fruit and vegetable drinks | 0 | 1 | 2 | 3 | 4 | 5 | A | B | C | D | E |  |
|  | Other soft drinks | 0 | 1 | 2 | 3 | 4 | 5 | A | B | C | D | E |  |
| 6. At supper | Tea | 0 | 1 | 2 | 3 | 4 | 5 | A | B | C | D | E |  |
|  | Water | 0 | 1 | 2 | 3 | 4 | 5 | A | B | C | D | E |  |
|  | Coffee | 0 | 1 | 2 | 3 | 4 | 5 | A | B | C | D | E |  |
|  | Milk and yogurt drink | 0 | 1 | 2 | 3 | 4 | 5 | A | B | C | D | E |  |
|  | Fruit and vegetable drinks | 0 | 1 | 2 | 3 | 4 | 5 | A | B | C | D | E |  |
|  | Other soft drinks | 0 | 1 | 2 | 3 | 4 | 5 | A | B | C | D | E |  |
| 7. After supper to 30 minutes before bedtime | Tea | 0 | 1 | 2 | 3 | 4 | 5 | A | B | C | D | E |  |
|  | Water | 0 | 1 | 2 | 3 | 4 | 5 | A | B | C | D | E |  |
|  | Coffee | 0 | 1 | 2 | 3 | 4 | 5 | A | B | C | D | E |  |
|  | Milk and yogurt drink | 0 | 1 | 2 | 3 | 4 | 5 | A | B | C | D | E |  |
|  | Fruit and vegetable drinks | 0 | 1 | 2 | 3 | 4 | 5 | A | B | C | D | E |  |
|  | Other soft drinks | 0 | 1 | 2 | 3 | 4 | 5 | A | B | C | D | E |  |
| 8. 30 minutes before bedtime to bedtime | Tea | 0 | 1 | 2 | 3 | 4 | 5 | A | B | C | D | E |  |
|  | water | 0 | 1 | 2 | 3 | 4 | 5 | A | B | C | D | E |  |
|  | Coffee | 0 | 1 | 2 | 3 | 4 | 5 | A | B | C | D | E |  |
|  | Milk and yogurt drink | 0 | 1 | 2 | 3 | 4 | 5 | A | B | C | D | E |  |
|  | Fruit and vegetable drinks | 0 | 1 | 2 | 3 | 4 | 5 | A | B | C | D | E |  |
|  | Other soft drinks | 0 | 1 | 2 | 3 | 4 | 5 | A | B | C | D | E |  |

◆ Water intake habits

| Your awareness of water intake | 1. Try to drink water even if you aren't thirsty | 2. Try to drink when you feel thirsty | 3. Not particularly conscious |
| --- | --- | --- | --- |
| How do you drink water | 1. Little by little | 2. Drink much at once |  |
| Your water intake compared to that of others | 1. More than others | 2. As much as anyone else. | 3. Less than others |
| Wake up and drink water while in bed | 1. Always | 2. Sometimes | 3. Never |
| Bring drinks from home when going out for long periods of time | 1. Always | 2. Sometimes | 3. Never |
| Put sugar in coffee and tea | 1. Always | 2. Sometimes | 3. Never |

◆ Alcohol intake

| Types of alcoholic beverages [definition of one drink]. | frequency | | | | | | amount | | | | | |
| --- | --- | --- | --- | --- | --- | --- | --- | --- | --- | --- | --- | --- |
|  | Do not drink | More than one day a month but less than one day a week | One day a week | 2 to 3 days a week | 4 to 6 days a week | Almost every day a week | Do not drink | 1 drink | 2 drinks | 3 drinks | 4 drinks or more |  |
| Beer [500 mL or 1 medium bottle or 1 beer mug] | 0 | 1 | 2 | 3 | 4 | 5 | A | B | C | D | E |  |
| Chu-hai and sour [350 mL] | 0 | 1 | 2 | 3 | 4 | 5 | A | B | C | D | E |  |
| Shochu, and Awamori [100 mL or 1 cup] | 0 | 1 | 2 | 3 | 4 | 5 | A | B | C | D | E |  |
| Sake [180 mL] | 0 | 1 | 2 | 3 | 4 | 5 | A | B | C | D | E |  |
| Whiskey, brandy, gin, vodka [30 mL or single cup] | 0 | 1 | 2 | 3 | 4 | 5 | A | B | C | D | E |  |
| Wine [120 mL or 1 glass] | 0 | 1 | 2 | 3 | 4 | 5 | A | B | C | D | E |  |

◆ Alcohol intake habits

| Have many opportunities to drink alcohol | 1. Yes | 2. No |
| --- | --- | --- |
| Drink alcohol during the day on non-working day | 1. Yes | 2. No |
| Eat too much when drinking alcohol | 1. Yes | 2. No |
| Eat low-calorie snacks when drinking alcohol | 1. Yes | 2. No |
| Not drink alcohol more than two days a week | 1. Yes | 2. No |

[Physical condition and constitution]

◆ Regarding your family (grandparents, parents, siblings)

| Someone in your family has/had diabetes | 1. Yes | 2. No |
| --- | --- | --- |
| Someone in your family is/was obesity | 1. Yes | 2. No |
| Someone in your family has/had dementia | 1. Yes | 2. No |
| Someone in your family has/had thinning hair | 1. Yes | 2. No |

◆ Physical condition

| Feel energetic | 1. Always | 2. Frequently | 3. Sometimes | 4. Seldom | 5. Never |
| --- | --- | --- | --- | --- | --- |
| Get tired easily | 1. Always | 2. Frequently | 3. Sometimes | 4. Seldom | 5. Never |
| Feel out of breath easily | 1. Always | 2. Frequently | 3. Sometimes | 4. Seldom | 5. Never |
| Feel your heart pounding | 1. Always | 2. Frequently | 3. Sometimes | 4. Seldom | 5. Never |
| Prefer a quiet environment and find it troublesome to talk to others | 1. Always | 2. Frequently | 3. Sometimes | 4. Seldom | 5. Never |
| Nausea and abdominal bloating | 1. Always | 2. Frequently | 3. Sometimes | 4. Seldom | 5. Never |
| Cold hands and feet even in warm places | 1. Always | 2. Frequently | 3. Sometimes | 4. Seldom | 5. Never |
| Coldness in areas other than hands and feet (back, abdomen, hips, knees, etc.) | 1. Always | 2. Frequently | 3. Sometimes | 4. Seldom | 5. Never |
| sweat even though not doing anything | 1. Always | 2. Frequently | 3. Sometimes | 4. Seldom | 5. Never |
| Greasy forehead | 1. Always | 2. Frequently | 3. Sometimes | 4. Seldom | 5. Never |
| Dry skin and lips | 1. Always | 2. Frequently | 3. Sometimes | 4. Seldom | 5. Never |
| Greasy nose | 1. Always | 2. Frequently | 3. Sometimes | 4. Seldom | 5. Never |
| Acne and pimples | 1. Always | 2. Frequently | 3. Sometimes | 4. Seldom | 5. Never |
| Sticky mouth | 1. Always | 2. Frequently | 3. Sometimes | 4. Seldom | 5. Never |
| Feel thirsty | 1. Always | 2. Frequently | 3. Sometimes | 4. Seldom | 5. Never |
| Diarrhea after intake cold food | 1. Always | 2. Frequently | 3. Sometimes | 4. Seldom | 5. Never |
| Sticky stool and feel of incomplete defecation | 1. Always | 2. Frequently | 3. Sometimes | 4. Seldom | 5. Never |
| Hard stool and constipation | 1. Always | 2. Frequently | 3. Sometimes | 4. Seldom | 5. Never |
| Bleed when brushing teeth | 1. Always | 2. Frequently | 3. Sometimes | 4. Seldom | 5. Never |
| Gingival recession and wide tooth gaps | 1. Always | 2. Frequently | 3. Sometimes | 4. Seldom | 5. Never |
| Food or drink stings your teeth. | 1. Always | 2. Frequently | 3. Sometimes | 4. Seldom | 5. Never |

◆Your constitution

| Easy to get fat | 1. Yes | 2. No |  |
| --- | --- | --- | --- |
| Hard to build muscle even after exercising | 1. Yes | 2. No |  |
| Cannot go through with a diet | 1. Yes | 2. No | 3 have never been on a diet |
| Regained weight after a diet | 1. Yes | 2. No | 3 have never been on a diet |
| Gained more than 10 kg after the age of 20 | 1. Yes | 2. No |  |
| Gained more than 10 kg after the age of 20 | 1. Yes | 2. No |  |
| Weight has changed by more than 3 kg in the last year | 1. Yes | 2. No |  |
| Go to the bathroom more often than others | 1. Yes | 2. No |  |
| Lower abdomen sticks out | 1. Yes | 2. No |  |
| Concerned about hair loss | 1. Yes | 2. No |  |
| Hair getting thinner | 1. Yes | 2. No |  |
| Flabby belly | 1. Yes | 2. No |  |
| Regular teeth | 1. Yes | 2. No |  |
| Tooth decay | 1. Yes | 2. No |  |
| Periodontal disease or alveolar pyorrhea | 1. Yes | 2. No |  |
| Often wear warm clothes in winter | 1. Yes | 2. No |  |
| Sensitive to cold | 1. Yes | 2. No |  |

[Workstyle and lifestyle]

◆ Workstyle

| Workstyle | 1. Full-time work | 2. Part-time work | 3. Other than 1, 2 or unemployed |  |  |  |
| --- | --- | --- | --- | --- | --- | --- |
| Do you have a night shift? | 1. Yes | 2. No | 3. Unemployed |  |  |  |
| Job description | 1. Sedentary work | 2. Standing work | 3. Physical work | 4. Unemployed |  |  |
| Requires complicated thinking | 1. Yes | 2. No | 3. Unemployed |  |  |  |
| Overtime in a month | 1. 80 hours or more | 2. 60 hours or more and less than 80 hours | 3. 40 hours or more and less than 60 hours | 4. 20 hours or more and less than 40 hours | 5 Less than 20 hours | 6. Unemployed |
| Often go home after 20:00 | 1. Yes | 2. No | 3. Unemployed |  |  |  |

◆ Oral hygiene habits

| Select all the times you brush your teeth | 1. After waking up | 2. After breakfast | 3. After lunch | 4. Within 1 hour after supper | 5. Before going to bed (after 1 hour after supper) |
| --- | --- | --- | --- | --- | --- |
| Use a dental floss or an interdental brush | 1. Every day | 2. 4-6 times a week | 3. 2-3 times a week | 4. Once a week | 5. Less than once a week |
| Use mouth rinse | 1. Every day | 2. 4-6 times a week | 3. 2-3 times a week | 4. Once a week | 5. Less than once a week |
| Frequency of toothbrush replacement | 1. Within 1 month | 2. Every 2 months | 3. Every 3 months | 4. Every 4 months | 5. Do not replace for more than 4 months |

◆ Lifestyle

| Walk faster than others | 1. Yes | 2. No |  |  |  |
| --- | --- | --- | --- | --- | --- |
| Often need to take stairs | 1. Yes | 2. No |  |  |  |
| Try to use stairs instead of elevator or escalator | 1. Yes | 2. No |  |  |  |
| Take time to soak in bath | 1. 30 min or more | 2. About 20 min | 3. About 10 min | 4. About 5 min | 5. shower only |
| Often lie down right after eating. | 1. Yes | 2. No |  |  |  |
| Often spend non-working days at home | 1. Yes | 2. No |  |  |  |

◆ Screen time (TV, computer, tablet, smart phone, etc.)

| On working day (if you don’t work, on weekdays) | 1. 7 hours or more | 2. 5 hours or more and less than 7 hours | 3. 3 hours or more and less than 5 hours | 4. 1 hour or more and less than 3 hours | 5. less than one hour |
| --- | --- | --- | --- | --- | --- |
| On non-working day (if you don’t work, on weekend) | 1. 7 hours or more | 2. 5 hours or more and less than 7 hours | 3. 3 hours or more and less than 5 hours | 4. 1 hour or more and less than 3 hours | 5. less than one hour |

◆ Please select all the benefits and target organs of supplements that you take at least 4 times a week.

| 1. Beauty and skin | 2. Health maintenance and improvement | 3. Joint | 4. Fatigue recovery | 5. Nutrition |
| --- | --- | --- | --- | --- |
| 6. Eye | 7. Antioxidant and anti- aging | 8. Weight loss | 9. Slimming (becomes slim with good style) | 10. Body fat suppression |
| 11. Stiffness and pain in the neck, shoulders, and back | 12. Bone | 13. High blood pressure | 14. Muscle | 15. Bowel control and constipation |
| 16. Anticoagulant | 17. Others | 18. Do not take supplements |  |  |

**Supplementary Table 1. Characteristics of the preprocessed questionnaire answers for each glycometabolic category**

|  | Category 1 | Category 2 | Category 3 | Category 4 |
| --- | --- | --- | --- | --- |
| ◆ Activity intensity | | | | |
| How did you spend your working days? | 1.83 (1.78–1.88) | 1.79 (1.71–1.86) | 1.89 (1.79–1.98) | 1.79 (1.71–1.86) |
| How did you spend your non-working days? | 1.51 (1.45–1.57) | 1.42 (1.35–1.50) | 1.51 (1.41–1.61) | 1.51 (1.43–1.59) |
| ◆ Travel method and time required (working days) | | | | |
| On foot | 3.10 (3.00–3.20) | 3.15 (3.02–3.27) | 3.14 (2.95–3.33) | 3.17 (3.03–3.32) |
| Running | 4.84 (4.79–4.89) | 4.84 (4.77–4.91) | 4.74 (4.63–4.85) | 4.81 (4.71–4.90) |
| By bicycle | 4.52 (4.44–4.59) | 4.55 (4.45–4.65) | 4.50 (4.35–4.64) | 4.54 (4.43–4.65) |
| By train / bus | 3.49 (3.37–3.61) | 3.52 (3.33–3.71) | 3.46 (3.22–3.70) | 3.51 (3.31–3.70) |
| By car / motorcycle | 4.65 (4.57–4.73) | 4.46 (4.31–4.61) | 4.57 (4.41–4.74) | 4.58 (4.45–4.71) |
| ◆ Travel method and time required (non-working days) | | | | |
| On foot | 3.48 (3.39–3.57) | 3.53 (3.40–3.66) | 3.61 (3.44–3.77) | 3.48 (3.34–3.62) |
| Running | 4.76 (4.70–4.82) | 4.80 (4.72–4.89) | 4.77 (4.67–4.88) | 4.78 (4.69–4.87) |
| By train / bus | 4.39 (4.31–4.47) | 4.33 (4.21–4.45) | 4.26 (4.08–4.45) | 4.35 (4.22–4.49) |
| By car / motorcycle | 4.50 (4.41–4.58) | 4.17 (4.00–4.33) | 4.37 (4.18–4.55) | 4.23 (4.06–4.39) |
| ◆ Duration of exercise (weekly total) | | | | |
| Walking | 4.00 (3.87–4.12) | 4.24 (4.08–4.39) | 4.10 (3.87–4.33) | 3.83 (3.62–4.04) |
| Swimming | 4.93 (4.89–4.97) | 4.96 (4.91–5.00) | 4.90 (4.82–4.99) | 4.94 (4.88–4.99) |
| Strength training | 4.52 (4.44–4.60) | 4.60 (4.49–4.71) | 4.61 (4.47–4.75) | 4.57 (4.44–4.71) |
| Other exercise | 4.43 (4.32–4.53) | 4.56 (4.43–4.70) | 4.59 (4.41–4.76) | 4.58 (4.44–4.71) |
| ◆ Please select the one that applies to your exercise situation and awareness of exercise. | | | | |
| You joined a sports gym | 1.87 (1.83–1.90) | 1.86 (1.82–1.91) | 1.86 (1.81–1.92) | 1.89 (1.84–1.93) |
| You own athletic shoes or gym clothes | 1.28 (1.24–1.32) | 1.32 (1.25–1.38) | 1.33 (1.25–1.41) | 1.29 (1.22–1.35) |
| Time of exercise (before meals) | 1.83 (1.80–1.87) | 1.88 (1.84–1.93) | 1.86 (1.80–1.92) | 1.86 (1.81–1.91) |
| Time of exercise (after meals) | 1.90 (1.87–1.93) | 1.92 (1.89–1.96) | 1.89 (1.83–1.94) | 1.93 (1.90–1.97) |
| Time of exercise (both before and after meals) | 1.98 (1.96–1.99) | 1.99 (1.98–2.00) | 1.98 (1.96–2.01) | 1.99 (1.97–2.00) |
| Time of exercise (not decided in particular) | 1.65 (1.61–1.70) | 1.67 (1.60–1.73) | 1.72 (1.64–1.80) | 1.59 (1.52–1.66) |
| Time of exercise (No exercise habits) | 1.63 (1.59–1.68) | 1.54 (1.47–1.61) | 1.55 (1.46–1.63) | 1.63 (1.56–1.70) |
| ◆ Sleep habits | | | | |
| Average sleeping time | 2.95 (2.86–3.03) | 2.85 (2.74–2.97) | 2.98 (2.84–3.11) | 2.88 (2.75–3.01) |
| Variation in bedtime | 1.31 (1.26–1.37) | 1.34 (1.25–1.43) | 1.26 (1.17–1.36) | 1.34 (1.25–1.43) |
| Falling asleep | 1.25 (1.21–1.29) | 1.29 (1.23–1.35) | 1.25 (1.17–1.32) | 1.25 (1.19–1.31) |
| Variation in sleep duration | 1.40 (1.34–1.45) | 1.43 (1.34–1.52) | 1.40 (1.29–1.51) | 1.35 (1.26–1.44) |
| ◆ Sleep habits and drowsiness | | | | |
| Feel that you get enough sleep | 2.54 (2.46–2.63) | 2.56 (2.41–2.70) | 2.67 (2.50–2.84) | 2.58 (2.42–2.74) |
| Stay up late | 3.27 (3.17–3.36) | 3.35 (3.20–3.51) | 3.53 (3.35–3.72) | 3.49 (3.32–3.66) |
| Take sleeping pills | 4.95 (4.93–4.98) | 4.90 (4.84–4.96) | 4.97 (4.93–5.01) | 4.93 (4.88–4.99) |
| Drink alcohol to sleep | 4.78 (4.72–4.84) | 4.75 (4.66–4.84) | 4.55 (4.37–4.73) | 4.63 (4.50–4.75) |
| You fall asleep and wake up right away | 4.40 (4.32–4.48) | 4.35 (4.23–4.47) | 4.33 (4.18–4.48) | 4.21 (4.06–4.35) |
| Frequency of mobile phone and tablet computer use at bedtime | 2.16 (2.06–2.26) | 2.09 (1.95–2.23) | 2.33 (2.13–2.53) | 2.27 (2.10–2.44) |
| Spend in a bright room until just before going to bed | 2.43 (2.31–2.55) | 2.44 (2.27–2.61) | 2.52 (2.29–2.74) | 2.42 (2.22–2.61) |
| Wake up in the middle of the night | 3.83 (3.73–3.92) | 3.68 (3.53–3.83) | 3.54 (3.35–3.74) | 3.49 (3.33–3.66) |
| Wake up during the night because you feel the need to go to the toilet. | 3.97 (3.87–4.07) | 3.87 (3.73–4.02) | 3.63 (3.42–3.84) | 3.63 (3.44–3.81) |
| Wake up in the middle of the night and have difficulty sleeping afterwards | 4.32 (4.25–4.40) | 4.28 (4.16–4.40) | 4.25 (4.09–4.40) | 4.16 (4.02–4.30) |
| Feel suffocation during sleep | 4.90 (4.87–4.93) | 4.90 (4.85–4.95) | 4.91 (4.86–4.96) | 4.81 (4.73–4.90) |
| When you wake up in the morning still feel sleepy and tired | 3.14 (3.03–3.24) | 3.23 (3.07–3.39) | 3.35 (3.18–3.53) | 3.28 (3.11–3.45) |
| Wake up refreshed in the morning | 2.84 (2.75–2.93) | 2.86 (2.70–3.02) | 2.74 (2.57–2.92) | 2.61 (2.46–2.76) |
| Feel sticky in the mouth when you wake up in the morning | 3.59 (3.48–3.70) | 3.70 (3.54–3.87) | 3.66 (3.46–3.87) | 3.47 (3.29–3.66) |
| Not hungry when you wake up in the morning. | 3.50 (3.39–3.61) | 3.50 (3.33–3.66) | 3.52 (3.32–3.72) | 3.44 (3.27–3.62) |
| Become drowsy after a meal | 2.82 (2.71–2.93) | 2.98 (2.83–3.13) | 2.97 (2.77–3.17) | 3.04 (2.88–3.19) |
| Long-lasting drowsiness after a meal | 3.48 (3.37–3.59) | 3.63 (3.47–3.78) | 3.76 (3.56–3.95) | 3.68 (3.52–3.83) |
| Wake up late on non-working day | 3.48 (3.38–3.59) | 3.47 (3.31–3.62) | 3.48 (3.28–3.68) | 3.69 (3.51–3.87) |
| You cannot wake up on non-working day without an alarm clock. | 4.03 (3.92–4.14) | 4.00 (3.84–4.17) | 4.21 (4.02–4.41) | 4.21 (4.03–4.38) |
| Take a nap on non-working day | 3.62 (3.51–3.72) | 3.54 (3.40–3.68) | 3.74 (3.56–3.91) | 3.49 (3.32–3.67) |
| Have habits to sleep well | 1.83 (1.79–1.86) | 1.86 (1.82–1.91) | 1.85 (1.79–1.91) | 1.81 (1.75–1.86) |
| Have been told that you have stopped breathing during sleep | 1.94 (1.91–1.96) | 1.89 (1.85–1.94) | 1.94 (1.90–1.98) | 1.88 (1.84–1.93) |
| Have been told you snores | 1.48 (1.43–1.52) | 1.40 (1.34–1.47) | 1.47 (1.38–1.55) | 1.30 (1.23–1.37) |
| Have been told you are bruxing your teeth during sleeping | 1.68 (1.63–1.72) | 1.69 (1.63–1.75) | 1.77 (1.70–1.85) | 1.68 (1.61–1.75) |
| ◆ Frequency and duration | | | | |
| Frequency of eating breakfast per week | 1.67 (1.57–1.78) | 1.57 (1.44–1.70) | 1.74 (1.55–1.94) | 1.57 (1.42–1.73) |
| Time required for breakfast | 3.25 (3.16–3.33) | 3.18 (3.06–3.31) | 3.15 (2.99–3.31) | 3.25 (3.12–3.38) |
| Frequency of eating lunch per week | 1.20 (1.15–1.25) | 1.16 (1.10–1.22) | 1.33 (1.20–1.46) | 1.23 (1.15–1.31) |
| Time required for lunch | 2.48 (2.40–2.56) | 2.39 (2.28–2.51) | 2.51 (2.37–2.66) | 2.52 (2.39–2.65) |
| Frequency of eating supper per week | 1.08 (1.05–1.12) | 1.08 (1.03–1.12) | 1.13 (1.04–1.22) | 1.16 (1.09–1.24) |
| Time required for supper | 1.97 (1.89–2.05) | 1.81 (1.70–1.92) | 1.86 (1.71–2.01) | 1.92 (1.79–2.04) |
| Frequency of eating after-supper snacks per week | 4.37 (4.27–4.47) | 4.35 (4.20–4.50) | 4.47 (4.30–4.64) | 4.49 (4.36–4.63) |
| Time required for after-supper snacks | 3.21 (3.14–3.28) | 3.06 (2.98–3.15) | 3.11 (2.98–3.23) | 3.13 (3.04–3.23) |
| Frequency of snacking per week (between breakfast and lunch or between lunch and dinner) | 3.40 (3.27–3.53) | 3.24 (3.04–3.44) | 3.56 (3.32–3.79) | 3.63 (3.43–3.82) |
| Time required for snacks (between breakfast and lunch or between lunch and dinner) | 4.07 (4.00–4.14) | 3.98 (3.87–4.09) | 4.05 (3.93–4.18) | 4.04 (3.95–4.13) |
| ◆ Meal timing | | | | |
| Eat breakfast at about the same time | 1.05 (1.03–1.07) | 1.09 (1.05–1.13) | 1.05 (1.01–1.08) | 1.08 (1.04–1.12) |
| Eat lunch at about the same time | 1.13 (1.10–1.16) | 1.11 (1.06–1.15) | 1.11 (1.05–1.16) | 1.13 (1.08–1.18) |
| Eat supper at about the same time | 1.19 (1.16–1.23) | 1.15 (1.10–1.20) | 1.16 (1.10–1.22) | 1.14 (1.09–1.19) |
| Eat after-supper snacks at about the same time | 1.13 (1.10–1.17) | 1.13 (1.08–1.17) | 1.14 (1.08–1.20) | 1.12 (1.07–1.17) |
| Eat snacks (between breakfast and lunch or between lunch and dinner) at about the same time | 1.31 (1.27–1.36) | 1.27 (1.21–1.33) | 1.29 (1.21–1.36) | 1.25 (1.19–1.31) |
| ◆ Dietary habits | | | | |
| Frequency of consuming yogurt and probiotics drinks | 3.70 (3.58–3.83) | 3.51 (3.32–3.71) | 3.63 (3.39–3.87) | 3.55 (3.34–3.75) |
| Which do you usually eat: rice or bread? | 1.99 (1.88–2.11) | 2.15 (1.97–2.33) | 1.81 (1.62–2.00) | 2.14 (1.95–2.32) |
| Amount of rice you usually ate compared to one serving of rice at a restaurant | 3.22 (3.12–3.31) | 3.17 (3.04–3.31) | 3.11 (2.92–3.29) | 3.21 (3.05–3.36) |
| Amount of main dish you usually ate compared to one serving of main dish at a restaurant | 3.01 (2.92–3.10) | 2.92 (2.80–3.04) | 2.81 (2.65–2.97) | 2.84 (2.71–2.97) |
| Frequency of soup intake | 3.14 (3.04–3.24) | 3.20 (3.07–3.33) | 3.01 (2.84–3.18) | 3.11 (2.96–3.26) |
| Frequency of noodles intake | 3.22 (3.12–3.31) | 3.13 (2.99–3.26) | 3.16 (2.99–3.33) | 3.18 (3.04–3.33) |
| Frequency of vegetable intake | 2.85 (2.76–2.95) | 2.93 (2.80–3.07) | 2.84 (2.67–3.01) | 2.79 (2.64–2.94) |
| Amount of soup left in noodle dish | 2.95 (2.83–3.08) | 3.06 (2.88–3.25) | 2.55 (2.32–2.78) | 2.87 (2.67–3.07) |
| ◆ Characteristics of your diet | | | | |
| Try to eat well-balanced diet | 1.24 (1.20–1.28) | 1.23 (1.17–1.29) | 1.18 (1.11–1.25) | 1.20 (1.14–1.26) |
| Try not to overeat | 1.46 (1.41–1.50) | 1.47 (1.40–1.53) | 1.49 (1.40–1.57) | 1.40 (1.33–1.47) |
| Try to choose low-carbohydrate diet | 1.68 (1.64–1.72) | 1.62 (1.55–1.69) | 1.65 (1.57–1.74) | 1.62 (1.55–1.69) |
| faster to eat than others | 1.54 (1.50–1.59) | 1.44 (1.37–1.51) | 1.54 (1.46–1.63) | 1.44 (1.37–1.51) |
| Try to choose low-salt diet | 1.54 (1.49–1.58) | 1.49 (1.42–1.56) | 1.61 (1.53–1.69) | 1.55 (1.48–1.62) |
| Try to eat vegetables first | 1.27 (1.23–1.31) | 1.31 (1.25–1.37) | 1.26 (1.19–1.34) | 1.26 (1.20–1.32) |
| Often eat sweets | 1.41 (1.36–1.45) | 1.41 (1.34–1.48) | 1.51 (1.43–1.60) | 1.51 (1.43–1.58) |
| Often eat seafood, tofu, and beans. | 1.40 (1.35–1.44) | 1.39 (1.33–1.46) | 1.35 (1.27–1.44) | 1.32 (1.25–1.39) |
| Eat more than other people | 1.64 (1.60–1.69) | 1.61 (1.54–1.67) | 1.63 (1.55–1.71) | 1.55 (1.48–1.62) |
| Often have a large serving and extra serving | 1.76 (1.72–1.80) | 1.78 (1.72–1.84) | 1.77 (1.69–1.84) | 1.76 (1.70–1.82) |
| Feel unsatisfied without rice dish or noodles after drinking alcohol | 1.83 (1.80–1.87) | 1.87 (1.82–1.92) | 1.81 (1.75–1.88) | 1.85 (1.80–1.90) |
| Finish supper two hours before going to bed | 1.29 (1.25–1.33) | 1.29 (1.23–1.35) | 1.28 (1.20–1.36) | 1.23 (1.17–1.29) |
| Meals served one by one | 1.28 (1.24–1.33) | 1.29 (1.22–1.35) | 1.22 (1.15–1.29) | 1.24 (1.18–1.30) |
| Often eat after-meal dessert | 1.20 (1.16–1.23) | 1.21 (1.16–1.27) | 1.22 (1.15–1.29) | 1.27 (1.21–1.34) |
| Often add salt or soy sauce to dishes. | 1.82 (1.79–1.86) | 1.84 (1.79–1.89) | 1.80 (1.74–1.87) | 1.76 (1.70–1.82) |
| Try to choose meat with less fat and remove excess fat. | 1.52 (1.48–1.57) | 1.54 (1.48–1.61) | 1.59 (1.51–1.68) | 1.58 (1.51–1.65) |
| Often eat until you're full. | 1.43 (1.38–1.47) | 1.35 (1.28–1.42) | 1.44 (1.35–1.52) | 1.48 (1.41–1.55) |
| Often eat rice dish or noodle or sweets after drinking alcohol. | 1.74 (1.70–1.78) | 1.76 (1.70–1.82) | 1.79 (1.72–1.86) | 1.77 (1.71–1.83) |
| Hardly ever cook at home | 1.65 (1.61–1.69) | 1.65 (1.58–1.72) | 1.72 (1.64–1.80) | 1.67 (1.61–1.74) |
| Try to chew well and eat | 1.48 (1.44–1.53) | 1.48 (1.41–1.55) | 1.55 (1.46–1.63) | 1.51 (1.43–1.58) |
| After supper, go to bed on a full stomach. | 1.75 (1.71–1.79) | 1.71 (1.65–1.78) | 1.71 (1.63–1.78) | 1.74 (1.68–1.80) |
| Try to eat a lot of vegetables | 1.35 (1.31–1.39) | 1.33 (1.27–1.40) | 1.32 (1.24–1.40) | 1.31 (1.24–1.37) |
| ◆ Meal details (days of intake per the week) | | | | |
| Rice ball | 1.83 (1.71–1.95) | 1.92 (1.74–2.11) | 1.80 (1.58–2.02) | 1.75 (1.57–1.94) |
| Rice porridge and risotto | 0.64 (0.57–0.71) | 0.70 (0.59–0.82) | 0.59 (0.46–0.71) | 0.69 (0.57–0.81) |
| Japanese seasoned rice with vegetables | 0.80 (0.73–0.87) | 0.83 (0.73–0.93) | 0.82 (0.70–0.94) | 0.84 (0.75–0.94) |
| Rice (white rice, brown rice, barley rice, etc.) | 4.24 (4.15–4.34) | 4.19 (4.04–4.33) | 4.35 (4.18–4.52) | 4.08 (3.93–4.24) |
| Fried rice, pilaf, and omelet rice | 1.34 (1.26–1.42) | 1.24 (1.13–1.36) | 1.26 (1.11–1.41) | 1.27 (1.15–1.39) |
| Sushi | 1.00 (0.94–1.07) | 1.01 (0.91–1.10) | 1.04 (0.91–1.16) | 0.94 (0.85–1.04) |
| Bowl of rice with topping | 1.38 (1.29–1.46) | 1.38 (1.26–1.51) | 1.37 (1.22–1.51) | 1.26 (1.14–1.38) |
| Curry rice, hashed beef rice | 1.31 (1.25–1.38) | 1.23 (1.14–1.33) | 1.40 (1.28–1.51) | 1.26 (1.16–1.36) |
| Plain bread | 2.22 (2.08–2.36) | 2.22 (2.01–2.44) | 2.13 (1.86–2.40) | 2.18 (1.95–2.40) |
| Bread rolls, croissants, French bread | 1.15 (1.05–1.24) | 1.17 (1.02–1.33) | 1.11 (0.94–1.27) | 1.18 (1.03–1.33) |
| Sweet bread, side dish bread, sandwiches, hamburgers, and pizza. | 2.03 (1.91–2.14) | 1.88 (1.72–2.04) | 1.83 (1.63–2.02) | 1.87 (1.69–2.04) |
| Noodles (ramen, udon, soba, pasta, etc.) | 2.11 (2.02–2.20) | 2.23 (2.08–2.37) | 2.28 (2.12–2.43) | 2.03 (1.89–2.18) |
| Okonomiyaki (savory Japanese-style of pancake.) | 0.78 (0.71–0.84) | 0.75 (0.65–0.85) | 0.73 (0.62–0.84) | 0.75 (0.64–0.85) |
| Cereal | 0.82 (0.70–0.94) | 0.79 (0.61–0.97) | 0.71 (0.50–0.93) | 0.69 (0.53–0.86) |
| Soup | 3.32 (3.18–3.45) | 3.09 (2.89–3.30) | 3.60 (3.38–3.83) | 3.39 (3.19–3.60) |
| Stew | 0.76 (0.70–0.83) | 0.69 (0.59–0.78) | 0.70 (0.59–0.80) | 0.73 (0.63–0.84) |
| Japanese hot pot dish | 1.03 (0.95–1.11) | 0.91 (0.78–1.04) | 1.03 (0.87–1.19) | 1.02 (0.89–1.14) |
| Simmered Food (main dishes: pot-au-feu, meat potato, etc.) | 1.87 (1.77–1.97) | 1.90 (1.73–2.06) | 1.86 (1.67–2.05) | 1.87 (1.71–2.04) |
| Simmered Food (side dishes: hijiki seaweed, dried strips of radish, etc.) | 1.79 (1.69–1.90) | 1.70 (1.54–1.86) | 1.68 (1.48–1.87) | 1.74 (1.57–1.92) |
| Deep-fried food | 2.14 (2.05–2.23) | 2.08 (1.94–2.21) | 2.23 (2.06–2.40) | 2.15 (2.02–2.29) |
| Grilled fish (grilled fish, grilled meat, hamburger steak, fried egg, etc.) | 2.57 (2.49–2.66) | 2.52 (2.39–2.66) | 2.80 (2.65–2.96) | 2.69 (2.55–2.83) |
| Stir-fried food (stir-fried vegetables, mapo tofu, roasted eggs, etc.) | 2.56 (2.47–2.64) | 2.45 (2.31–2.59) | 2.56 (2.41–2.71) | 2.47 (2.34–2.60) |
| Boiled and steamed food (hot vegetables, boiled eggs, chawanmushi, shumai, etc.) | 1.91 (1.81–2.01) | 1.90 (1.74–2.06) | 2.08 (1.89–2.28) | 1.88 (1.71–2.04) |
| Raw food (sashimi, natto, tofu, cod roe, etc.) | 2.43 (2.32–2.55) | 2.46 (2.29–2.63) | 2.63 (2.41–2.85) | 2.42 (2.25–2.59) |
| Salad, raw vegetables | 3.29 (3.18–3.40) | 3.25 (3.08–3.43) | 3.44 (3.25–3.64) | 3.35 (3.17–3.54) |
| Vinegared food | 1.18 (1.08–1.28) | 1.21 (1.05–1.37) | 1.26 (1.06–1.46) | 1.32 (1.16–1.48) |
| Ohitashi (boiled spinach seasoned with soy sauce) | 1.32 (1.22–1.43) | 1.27 (1.12–1.43) | 1.32 (1.15–1.50) | 1.30 (1.14–1.46) |
| Japanese pickles | 1.61 (1.49–1.73) | 1.59 (1.41–1.77) | 1.68 (1.44–1.93) | 1.61 (1.41–1.81) |
| Cheese, dried fruits, nuts | 2.44 (2.30–2.57) | 2.57 (2.35–2.79) | 2.49 (2.22–2.76) | 2.47 (2.27–2.67) |
| Fruits (except berries) | 1.99 (1.86–2.11) | 1.98 (1.77–2.19) | 1.76 (1.53–1.99) | 2.07 (1.87–2.27) |
| Fruits (berries) | 1.35 (1.23–1.46) | 1.43 (1.27–1.60) | 1.25 (1.05–1.45) | 1.35 (1.19–1.51) |
| Yogurt, pudding, jelly, ice cream | 2.41 (2.27–2.54) | 2.44 (2.24–2.64) | 2.32 (2.05–2.60) | 2.46 (2.24–2.68) |
| Cakes, pies, tarts | 1.19 (1.11–1.27) | 1.16 (1.04–1.28) | 1.12 (0.96–1.28) | 1.02 (0.91–1.13) |
| Traditional Japanese sweets | 1.11 (1.03–1.18) | 1.13 (1.00–1.25) | 1.07 (0.91–1.23) | 1.11 (0.98–1.24) |
| Fried confectionery | 2.14 (2.02–2.25) | 2.27 (2.10–2.44) | 2.18 (1.96–2.40) | 2.10 (1.90–2.30) |
| ◆ Drink intake (after waking up-before breakfast) | | | | |
| Tea (frequency) | 1.78 (1.59–1.96) | 1.85 (1.57–2.14) | 1.59 (1.23–1.94) | 1.69 (1.39–1.99) |
| Water (frequency) | 3.03 (2.83–3.22) | 3.19 (2.90–3.47) | 3.05 (2.69–3.42) | 3.23 (2.92–3.53) |
| Coffee (frequency) | 1.81 (1.61–2.01) | 2.05 (1.75–2.34) | 1.87 (1.50–2.25) | 1.84 (1.54–2.14) |
| Milk and yogurt drink (frequency) | 1.29 (1.13–1.45) | 1.21 (0.99–1.44) | 0.92 (0.66–1.18) | 1.10 (0.87–1.33) |
| Fruit and vegetable drinks (frequency) | 0.77 (0.65–0.88) | 0.86 (0.67–1.05) | 0.56 (0.37–0.74) | 0.82 (0.62–1.02) |
| Other soft drinks (frequency) | 0.66 (0.55–0.77) | 0.64 (0.48–0.80) | 0.62 (0.42–0.83) | 0.54 (0.38–0.69) |
| ◆ Drink intake (at breakfast) | | | | |
| Tea (frequency) | 1.82 (1.63–2.01) | 1.97 (1.68–2.25) | 1.86 (1.51–2.21) | 1.82 (1.52–2.12) |
| Water (frequency) | 2.11 (1.91–2.32) | 2.14 (1.83–2.44) | 1.92 (1.56–2.29) | 2.03 (1.72–2.35) |
| Coffee (frequency) | 2.23 (2.03–2.43) | 2.42 (2.13–2.72) | 2.44 (2.07–2.81) | 2.33 (2.03–2.62) |
| Milk and yogurt drink (frequency) | 1.39 (1.22–1.55) | 1.53 (1.29–1.78) | 1.38 (1.08–1.67) | 1.35 (1.12–1.59) |
| Fruit and vegetable drinks (frequency) | 0.79 (0.67–0.91) | 0.90 (0.71–1.09) | 0.68 (0.48–0.89) | 0.99 (0.78–1.20) |
| Other soft drinks (frequency) | 0.40 (0.31–0.50) | 0.43 (0.29–0.56) | 0.38 (0.22–0.54) | 0.44 (0.28–0.59) |
| ◆ Drink intake (after breakfast-before lunch) | | | | |
| Tea (frequency) | 2.00 (1.82–2.19) | 2.28 (2.00–2.56) | 1.94 (1.60–2.28) | 2.29 (2.00–2.59) |
| Water (frequency) | 2.40 (2.20–2.60) | 2.37 (2.08–2.66) | 2.24 (1.88–2.60) | 2.46 (2.15–2.77) |
| Coffee (frequency) | 2.06 (1.87–2.24) | 2.02 (1.75–2.30) | 2.10 (1.74–2.45) | 2.01 (1.72–2.29) |
| Milk and yogurt drink (frequency) | 0.62 (0.50–0.74) | 0.59 (0.41–0.76) | 0.49 (0.29–0.69) | 0.66 (0.49–0.84) |
| Fruit and vegetable drinks (frequency) | 0.44 (0.35–0.54) | 0.50 (0.36–0.64) | 0.37 (0.23–0.50) | 0.59 (0.43–0.75) |
| Other soft drinks (frequency) | 0.50 (0.40–0.60) | 0.56 (0.41–0.71) | 0.64 (0.44–0.84) | 0.69 (0.51–0.87) |
| ◆ Drink intake (at lunch) | | | | |
| Tea (frequency) | 2.71 (2.53–2.88) | 3.08 (2.82–3.35) | 2.92 (2.60–3.25) | 2.82 (2.55–3.09) |
| Tea (amount) | 1.55 (1.44–1.65) | 1.72 (1.56–1.88) | 1.70 (1.51–1.89) | 1.56 (1.40–1.73) |
| Water (frequency) | 2.27 (2.08–2.45) | 2.39 (2.11–2.67) | 2.21 (1.88–2.55) | 2.29 (2.01–2.58) |
| Coffee (frequency) | 1.37 (1.21–1.53) | 1.40 (1.15–1.64) | 1.41 (1.13–1.70) | 1.29 (1.06–1.53) |
| Milk and yogurt drink (frequency) | 0.49 (0.39–0.60) | 0.49 (0.34–0.64) | 0.53 (0.34–0.71) | 0.61 (0.44–0.78) |
| Fruit and vegetable drinks (frequency) | 0.49 (0.40–0.58) | 0.45 (0.31–0.58) | 0.48 (0.31–0.65) | 0.62 (0.46–0.78) |
| Other soft drinks (frequency) | 0.49 (0.40–0.59) | 0.49 (0.34–0.63) | 0.49 (0.32–0.65) | 0.57 (0.42–0.73) |
| ◆ Drink intake (after lunch-before dinner) | | | | |
| Tea (frequency) | 2.29 (2.12–2.47) | 2.51 (2.25–2.78) | 2.15 (1.82–2.48) | 2.38 (2.12–2.65) |
| Tea (amount) | 1.41 (1.30–1.51) | 1.55 (1.39–1.72) | 1.35 (1.14–1.57) | 1.53 (1.35–1.70) |
| Water (frequency) | 2.29 (2.10–2.48) | 2.42 (2.14–2.70) | 2.37 (2.02–2.71) | 2.34 (2.04–2.63) |
| Coffee (frequency) | 2.23 (2.06–2.41) | 2.22 (1.96–2.48) | 2.38 (2.05–2.72) | 2.11 (1.83–2.39) |
| Milk and yogurt drink (frequency) | 0.58 (0.47–0.68) | 0.58 (0.41–0.75) | 0.41 (0.25–0.58) | 0.54 (0.38–0.70) |
| Fruit and vegetable drinks (frequency) | 0.44 (0.37–0.52) | 0.48 (0.35–0.60) | 0.41 (0.27–0.56) | 0.53 (0.38–0.67) |
| Other soft drinks (frequency) | 0.72 (0.61–0.83) | 0.70 (0.54–0.86) | 0.76 (0.55–0.97) | 0.78 (0.60–0.96) |
| ◆ Drink intake (at supper) | | | | |
| Tea (frequency) | 2.42 (2.23–2.61) | 2.73 (2.45–3.02) | 2.58 (2.20–2.95) | 2.41 (2.10–2.71) |
| Water (frequency) | 2.03 (1.84–2.22) | 2.13 (1.85–2.42) | 2.03 (1.68–2.38) | 2.05 (1.75–2.34) |
| Coffee (frequency) | 0.47 (0.36–0.58) | 0.47 (0.30–0.64) | 0.38 (0.19–0.56) | 0.58 (0.40–0.77) |
| Milk and yogurt drink (frequency) | 0.29 (0.22–0.37) | 0.28 (0.17–0.40) | 0.26 (0.12–0.39) | 0.35 (0.21–0.49) |
| Fruit and vegetable drinks (frequency) | 0.31 (0.24–0.39) | 0.34 (0.21–0.48) | 0.31 (0.16–0.46) | 0.35 (0.23–0.47) |
| Other soft drinks (frequency) | 0.52 (0.42–0.63) | 0.44 (0.29–0.58) | 0.48 (0.30–0.66) | 0.54 (0.37–0.70) |
| ◆ Drink intake (after supper to 30 minutes before bedtime) | | | | |
| Tea (frequency) | 1.98 (1.79–2.16) | 1.87 (1.61–2.13) | 1.78 (1.43–2.13) | 1.68 (1.40–1.96) |
| Water (frequency) | 2.49 (2.30–2.68) | 2.61 (2.32–2.90) | 2.14 (1.77–2.50) | 2.38 (2.08–2.69) |
| Coffee (frequency) | 0.93 (0.78–1.07) | 0.99 (0.76–1.22) | 0.86 (0.60–1.12) | 0.99 (0.74–1.23) |
| Milk and yogurt drink (frequency) | 0.56 (0.46–0.67) | 0.62 (0.44–0.79) | 0.37 (0.21–0.53) | 0.48 (0.33–0.63) |
| Fruit and vegetable drinks (frequency) | 0.37 (0.29–0.44) | 0.35 (0.23–0.48) | 0.35 (0.21–0.49) | 0.36 (0.24–0.49) |
| Other soft drinks (frequency) | 0.56 (0.46–0.67) | 0.48 (0.34–0.62) | 0.62 (0.42–0.83) | 0.59 (0.43–0.75) |
| ◆ Drink intake (30 minutes before bedtime to bedtime) | | | | |
| Tea (frequency) | 0.85 (0.70–1.00) | 0.74 (0.54–0.95) | 0.94 (0.65–1.23) | 0.67 (0.46–0.88) |
| Water (frequency) | 2.04 (1.85–2.24) | 2.06 (1.77–2.35) | 2.06 (1.69–2.43) | 2.13 (1.82–2.43) |
| Coffee (frequency) | 0.27 (0.19–0.35) | 0.25 (0.13–0.38) | 0.16 (0.04–0.28) | 0.28 (0.15–0.42) |
| Milk and yogurt drink (frequency) | 0.27 (0.18–0.35) | 0.17 (0.08–0.27) | 0.14 (0.05–0.23) | 0.24 (0.12–0.36) |
| Fruit and vegetable drinks (frequency) | 0.13 (0.08–0.17) | 0.11 (0.04–0.17) | 0.17 (0.06–0.27) | 0.16 (0.07–0.24) |
| Other soft drinks (frequency) | 0.25 (0.18–0.32) | 0.22 (0.12–0.32) | 0.31 (0.14–0.47) | 0.23 (0.12–0.34) |
| ◆ Water intake habits | | | | |
| Try to drink water even if you aren't thirsty | 1.57 (1.52–1.62) | 1.57 (1.50–1.64) | 1.64 (1.56–1.72) | 1.54 (1.47–1.61) |
| Try to drink when you feel thirsty | 1.64 (1.59–1.68) | 1.63 (1.56–1.70) | 1.65 (1.56–1.73) | 1.67 (1.61–1.74) |
| Not particularly conscious | 1.79 (1.75–1.83) | 1.80 (1.75–1.86) | 1.71 (1.64–1.79) | 1.79 (1.73–1.85) |
| How do you drink water | 1.25 (1.21–1.29) | 1.24 (1.18–1.30) | 1.32 (1.24–1.40) | 1.29 (1.22–1.35) |
| Your water intake compared to that of others | 1.90 (1.83–1.96) | 1.95 (1.86–2.04) | 1.85 (1.73–1.97) | 1.74 (1.65–1.84) |
| Wake up and drink water while in bed | 2.70 (2.65–2.75) | 2.77 (2.69–2.84) | 2.60 (2.49–2.71) | 2.62 (2.53–2.71) |
| Bring drinks from home when going out for long periods of time | 1.85 (1.78–1.92) | 1.84 (1.73–1.96) | 1.89 (1.74–2.03) | 1.81 (1.69–1.92) |
| Put sugar in coffee and tea | 2.42 (2.35–2.49) | 2.41 (2.31–2.52) | 2.29 (2.14–2.43) | 2.51 (2.40–2.62) |
| ◆ Alcohol intake | | | | |
| Beer (frequency) | 1.06 (0.93–1.18) | 0.97 (0.78–1.15) | 1.29 (1.02–1.57) | 1.26 (1.04–1.48) |
| Chu-hai and sour (frequency) | 0.89 (0.78–1.00) | 0.82 (0.66–0.98) | 1.20 (0.93–1.46) | 0.98 (0.78–1.17) |
| Shochu, and Awamori (frequency) | 0.25 (0.19–0.32) | 0.15 (0.07–0.23) | 0.38 (0.21–0.56) | 0.53 (0.37–0.69) |
| Sake (frequency) | 0.26 (0.20–0.32) | 0.22 (0.13–0.30) | 0.19 (0.09–0.29) | 0.24 (0.15–0.32) |
| Whiskey, brandy, gin, vodka (frequency) | 0.22 (0.16–0.28) | 0.12 (0.06–0.18) | 0.31 (0.15–0.47) | 0.29 (0.18–0.40) |
| Wine (frequency) | 0.51 (0.43–0.60) | 0.52 (0.39–0.66) | 0.42 (0.27–0.57) | 0.49 (0.37–0.62) |
| ◆ Alcohol intake habits | | | | |
| Have many opportunities to drink alcohol | 1.82 (1.78–1.85) | 1.82 (1.76–1.87) | 1.75 (1.68–1.83) | 1.76 (1.70–1.82) |
| Drink alcohol during the day on non-working day | 1.92 (1.89–1.94) | 1.87 (1.82–1.92) | 1.88 (1.82–1.94) | 1.88 (1.83–1.93) |
| Eat too much when drinking alcohol | 1.87 (1.84–1.90) | 1.83 (1.78–1.89) | 1.83 (1.76–1.89) | 1.86 (1.81–1.91) |
| Eat low-calorie snacks when drinking alcohol | 1.67 (1.62–1.71) | 1.68 (1.62–1.75) | 1.78 (1.71–1.85) | 1.65 (1.58–1.72) |
| Not drink alcohol more than two days a week | 1.23 (1.19–1.26) | 1.23 (1.17–1.29) | 1.35 (1.26–1.43) | 1.21 (1.15–1.27) |
| ◆ Regarding your family (grandparents, parents, siblings) | | | | |
| Someone in your family has/had diabetes | 1.73 (1.69–1.77) | 1.63 (1.56–1.70) | 1.76 (1.69–1.83) | 1.66 (1.60–1.73) |
| Someone in your family is/was obesity | 1.77 (1.73–1.81) | 1.77 (1.71–1.83) | 1.74 (1.66–1.81) | 1.72 (1.66–1.79) |
| Someone in your family has/had dementia | 1.85 (1.81–1.88) | 1.82 (1.77–1.87) | 1.86 (1.81–1.92) | 1.79 (1.74–1.85) |
| Someone in your family has/had thinning hair | 1.66 (1.61–1.70) | 1.65 (1.58–1.72) | 1.54 (1.46–1.63) | 1.62 (1.55–1.69) |
| ◆ Physical condition | | | | |
| Feel energetic | 2.13 (2.05–2.22) | 2.16 (2.03–2.28) | 2.20 (2.04–2.35) | 2.20 (2.07–2.33) |
| Get tired easily | 3.23 (3.14–3.33) | 3.14 (3.00–3.28) | 3.49 (3.32–3.66) | 3.39 (3.24–3.55) |
| Feel out of breath easily | 4.52 (4.45–4.60) | 4.29 (4.15–4.42) | 4.53 (4.39–4.68) | 4.35 (4.22–4.49) |
| Feel your heart pounding | 4.65 (4.59–4.71) | 4.60 (4.50–4.71) | 4.67 (4.55–4.79) | 4.64 (4.54–4.74) |
| Prefer a quiet environment and find it troublesome to talk to others | 3.70 (3.59–3.81) | 3.61 (3.45–3.77) | 4.01 (3.83–4.18) | 3.86 (3.70–4.02) |
| Nausea and abdominal bloating | 4.34 (4.26–4.42) | 4.18 (4.05–4.32) | 4.52 (4.39–4.65) | 4.27 (4.14–4.40) |
| Cold hands and feet even in warm places | 4.35 (4.25–4.45) | 4.31 (4.16–4.45) | 4.52 (4.36–4.68) | 4.55 (4.41–4.69) |
| Coldness in areas other than hands and feet (back, abdomen, hips, knees, etc.) | 4.44 (4.35–4.54) | 4.41 (4.28–4.54) | 4.59 (4.43–4.76) | 4.57 (4.44–4.69) |
| sweat even though not doing anything | 4.63 (4.56–4.71) | 4.59 (4.49–4.68) | 4.71 (4.60–4.81) | 4.51 (4.37–4.64) |
| Greasy forehead | 4.16 (4.06–4.27) | 3.83 (3.67–3.99) | 4.12 (3.92–4.32) | 3.91 (3.73–4.08) |
| Dry skin and lips | 3.66 (3.55–3.77) | 3.71 (3.56–3.86) | 3.60 (3.39–3.82) | 3.73 (3.54–3.91) |
| Greasy nose | 3.77 (3.66–3.88) | 3.37 (3.19–3.55) | 3.74 (3.53–3.96) | 3.63 (3.45–3.81) |
| Acne and pimples | 4.13 (4.03–4.23) | 4.00 (3.85–4.14) | 4.32 (4.15–4.49) | 4.29 (4.16–4.43) |
| Feel thirsty | 3.57 (3.48–3.66) | 3.45 (3.31–3.60) | 3.61 (3.44–3.78) | 3.62 (3.48–3.77) |
| Diarrhea after intake cold food | 4.46 (4.37–4.54) | 4.37 (4.24–4.50) | 4.39 (4.24–4.54) | 4.52 (4.41–4.64) |
| Sticky stool and feel of incomplete defecation | 4.42 (4.34–4.49) | 4.36 (4.23–4.49) | 4.58 (4.46–4.70) | 4.42 (4.30–4.53) |
| Hard stool and constipation | 4.02 (3.91–4.12) | 3.94 (3.77–4.10) | 4.11 (3.92–4.29) | 4.28 (4.14–4.43) |
| Bleed when brushing teeth | 4.15 (4.06–4.24) | 4.09 (3.96–4.23) | 4.21 (4.03–4.39) | 4.19 (4.05–4.34) |
| Gingival recession and wide tooth gaps | 3.96 (3.84–4.07) | 3.96 (3.79–4.13) | 3.99 (3.78–4.20) | 3.86 (3.67–4.05) |
| Food or drink stings your teeth. | 4.29 (4.20–4.37) | 4.22 (4.07–4.37) | 4.49 (4.35–4.62) | 4.24 (4.09–4.39) |
| ◆Your constitution | | | | |
| Easy to get fat | 1.51 (1.47–1.56) | 1.30 (1.24–1.36) | 1.56 (1.48–1.65) | 1.39 (1.32–1.46) |
| Hard to build muscle even after exercising | 1.56 (1.51–1.60) | 1.50 (1.43–1.56) | 1.59 (1.51–1.68) | 1.53 (1.46–1.60) |
| Have been on a diet | 1.35 (1.31–1.39) | 1.24 (1.18–1.30) | 1.41 (1.32–1.49) | 1.32 (1.25–1.39) |
| Cannot go through with a diet | 1.25 (1.21–1.29) | 1.17 (1.12–1.22) | 1.20 (1.13–1.27) | 1.16 (1.11–1.22) |
| Regained weight after a diet | 1.26 (1.22–1.30) | 1.22 (1.16–1.28) | 1.23 (1.16–1.31) | 1.18 (1.13–1.24) |
| Gained more than 10 kg after the age of 20 | 1.73 (1.68–1.77) | 1.48 (1.41–1.54) | 1.66 (1.58–1.74) | 1.48 (1.41–1.55) |
| Gained more than 10 kg after the age of 20 | 1.62 (1.58–1.67) | 1.52 (1.46–1.59) | 1.64 (1.56–1.72) | 1.59 (1.52–1.66) |
| Weight has changed by more than 3 kg in the last year | 1.38 (1.34–1.43) | 1.37 (1.31–1.44) | 1.36 (1.28–1.44) | 1.27 (1.21–1.34) |
| Go to the bathroom more often than others | 1.59 (1.54–1.63) | 1.59 (1.52–1.66) | 1.54 (1.46–1.63) | 1.52 (1.45–1.59) |
| Lower abdomen sticks out | 1.56 (1.52–1.61) | 1.41 (1.34–1.48) | 1.44 (1.36–1.53) | 1.47 (1.40–1.55) |
| Concerned about hair loss | 1.69 (1.65–1.73) | 1.61 (1.54–1.68) | 1.62 (1.54–1.71) | 1.52 (1.45–1.59) |
| Hair getting thinner | 1.67 (1.63–1.71) | 1.58 (1.51–1.65) | 1.54 (1.46–1.63) | 1.50 (1.43–1.57) |
| Flabby belly | 1.54 (1.50–1.59) | 1.33 (1.27–1.39) | 1.50 (1.42–1.59) | 1.36 (1.29–1.43) |
| Regular teeth | 1.48 (1.43–1.52) | 1.46 (1.39–1.53) | 1.49 (1.40–1.57) | 1.52 (1.45–1.59) |
| Tooth decay | 1.90 (1.87–1.93) | 1.94 (1.91–1.97) | 1.89 (1.84–1.95) | 1.95 (1.92–1.98) |
| Periodontal disease or alveolar pyorrhea | 1.79 (1.75–1.83) | 1.78 (1.72–1.83) | 1.71 (1.63–1.78) | 1.73 (1.66–1.79) |
| Often wear warm clothes in winter | 1.62 (1.58–1.67) | 1.66 (1.59–1.72) | 1.65 (1.57–1.74) | 1.73 (1.67–1.79) |
| Sensitive to cold | 1.49 (1.44–1.54) | 1.56 (1.49–1.63) | 1.57 (1.49–1.66) | 1.63 (1.56–1.70) |
| ◆ Workstyle | | | | |
| Workstyle (full-time work) | 1.39 (1.35–1.44) | 1.44 (1.37–1.51) | 1.36 (1.28–1.44) | 1.43 (1.36–1.50) |
| Workstyle (part-time work) | 1.77 (1.73–1.81) | 1.70 (1.64–1.77) | 1.78 (1.71–1.85) | 1.75 (1.69–1.81) |
| Did you work? | 1.14 (1.11–1.17) | 1.14 (1.09–1.18) | 1.13 (1.07–1.19) | 1.17 (1.11–1.22) |
| Did you have a night shift? | 1.96 (1.94–1.98) | 1.97 (1.95–1.99) | 1.98 (1.96–2.01) | 1.97 (1.95–2.00) |
| Job description | 1.40 (1.34–1.46) | 1.32 (1.23–1.40) | 1.40 (1.28–1.52) | 1.36 (1.27–1.45) |
| Requires complicated thinking | 1.60 (1.56–1.65) | 1.57 (1.50–1.64) | 1.61 (1.53–1.69) | 1.58 (1.51–1.65) |
| Overtime in a month | 4.67 (4.60–4.74) | 4.62 (4.50–4.74) | 4.64 (4.51–4.77) | 4.65 (4.53–4.76) |
| Often go home after 20:00 | 1.70 (1.66–1.74) | 1.75 (1.69–1.81) | 1.71 (1.64–1.79) | 1.76 (1.70–1.82) |
| ◆ Oral hygiene habits | | | | |
| Select all the times you brush your teeth (after waking up) | 1.63 (1.58–1.67) | 1.61 (1.54–1.67) | 1.53 (1.45–1.62) | 1.55 (1.48–1.62) |
| Select all the times you brush your teeth (after breakfast) | 1.40 (1.35–1.44) | 1.46 (1.39–1.53) | 1.50 (1.42–1.59) | 1.42 (1.35–1.49) |
| Select all the times you brush your teeth (after lunch) | 1.58 (1.53–1.63) | 1.67 (1.61–1.74) | 1.67 (1.59–1.75) | 1.62 (1.55–1.69) |
| Select all the times you brush your teeth (within 1 hour after supper) | 1.77 (1.74–1.81) | 1.82 (1.76–1.87) | 1.81 (1.75–1.88) | 1.75 (1.69–1.81) |
| Select all the times you brush your teeth (Before going to bed) | 1.23 (1.19–1.27) | 1.24 (1.18–1.30) | 1.25 (1.17–1.32) | 1.32 (1.25–1.39) |
| Use a dental floss or an interdental brush | 3.82 (3.68–3.96) | 3.58 (3.36–3.80) | 3.61 (3.32–3.90) | 3.69 (3.46–3.92) |
| Use mouth rinse | 4.01 (3.87–4.15) | 4.00 (3.79–4.20) | 3.83 (3.56–4.10) | 3.77 (3.54–4.01) |
| Frequency of toothbrush replacement | 2.61 (2.50–2.72) | 2.67 (2.50–2.84) | 2.52 (2.32–2.72) | 2.45 (2.28–2.61) |
| ◆ Lifestyle | | | | |
| Walk faster than others | 1.33 (1.29–1.38) | 1.43 (1.36–1.50) | 1.37 (1.29–1.45) | 1.34 (1.27–1.40) |
| Often need to take stairs | 1.52 (1.48–1.57) | 1.58 (1.51–1.65) | 1.59 (1.50–1.67) | 1.58 (1.51–1.65) |
| Try to use stairs instead of elevator or escalator | 1.56 (1.51–1.61) | 1.55 (1.48–1.62) | 1.56 (1.48–1.65) | 1.58 (1.51–1.65) |
| Take time to soak in bath | 3.19 (3.06–3.32) | 3.11 (2.94–3.29) | 3.32 (3.10–3.55) | 3.47 (3.28–3.66) |
| Often lie down right after eating. | 1.60 (1.55–1.64) | 1.56 (1.49–1.63) | 1.64 (1.56–1.72) | 1.65 (1.58–1.72) |
| Often spend non-working days at home | 1.60 (1.56–1.65) | 1.58 (1.51–1.65) | 1.60 (1.52–1.69) | 1.52 (1.45–1.59) |
| ◆ Screen time (TV, computer, tablet, smart phone, etc.) | | | | |
| On working day (if you don’t work, on weekdays) | 2.64 (2.53–2.75) | 2.42 (2.25–2.59) | 2.52 (2.31–2.73) | 2.66 (2.48–2.83) |
| On non-working day (if you don’t work, on weekend) | 2.76 (2.67–2.86) | 2.55 (2.40–2.70) | 2.68 (2.50–2.85) | 2.76 (2.60–2.93) |
| ◆ Please select all the benefits and target organs of supplements that you take at least 4 times a week. | | | | |
| Beauty and skin | 1.94 (1.92–1.96) | 1.92 (1.88–1.96) | 1.96 (1.93–2.00) | 1.92 (1.88–1.96) |
| Health maintenance and improvement | 1.90 (1.87–1.93) | 1.96 (1.93–1.98) | 1.91 (1.86–1.96) | 1.91 (1.87–1.95) |
| Joint | 1.99 (1.99–2.00) | 2.00 (2.00–2.00) | 1.99 (1.98–2.01) | 1.97 (1.94–1.99) |
| Fatigue recovery | 1.95 (1.93–1.97) | 1.97 (1.95–1.99) | 1.93 (1.89–1.98) | 1.97 (1.95–2.00) |
| Nutrition | 1.94 (1.91–1.96) | 1.97 (1.94–1.99) | 1.94 (1.90–1.98) | 1.90 (1.86–1.94) |
| Eye | 1.98 (1.97–1.99) | 1.99 (1.98–2.00) | 1.99 (1.98–2.01) | 1.97 (1.95–2.00) |
| Antioxidant and anti- aging | 1.97 (1.96–1.99) | 1.99 (1.98–2.00) | 1.98 (1.95–2.00) | 1.98 (1.96–2.00) |
| Weight loss | 2.00 (1.99–2.00) | 1.99 (1.98–2.00) | 1.98 (1.96–2.01) | 1.99 (1.98–2.01) |
| Slimming (becomes slim with good style) | 1.99 (1.98–2.00) | 2.00 (2.00–2.00) | 1.99 (1.98–2.01) | 1.99 (1.98–2.01) |
| Body fat suppression | 1.99 (1.99–2.00) | 1.98 (1.95–2.00) | 1.98 (1.96–2.01) | 1.99 (1.97–2.00) |
| Stiffness and pain in the neck, shoulders, and back | 2 .00(1.99–2.00) | 2.00 (2.00–2.00) | 1.99 (1.98–2.01) | 1.99 (1.98–2.01) |
| Bone | 2 .00(1.99–2.00) | 2.00 (2.00–2.00) | 1.99 (1.98–2.01) | 1.99 (1.97–2.00) |
| High blood pressure | 2.00 (2.00–2.00) | 2.00 (2.00–2.00) | 1.99 (1.98–2.01) | 1.99 (1.97–2.00) |
| Muscle | 1.98 (1.96–1.99) | 1.99 (1.97–2.00) | 1.98 (1.95–2.00) | 1.98 (1.97–2.00) |
| Bowel control and constipation | 1.98 (1.97–1.99) | 1.99 (1.97–2.00) | 1.98 (1.96–2.01) | 1.96 (1.93–1.99) |
| Anticoagulant | 1.99 (1.98–2.00) | 1.99 (1.98–2.00) | 1.99 (1.98–2.01) | 1.99 (1.98–2.01) |
| Others | 1.99 (1.98–2.00) | 2.00 (1.99–2.00) | 2.00 (2.00–2.00) | 1.99 (1.98–2.01) |
| Do not take supplements | 1.16 (1.13–1.20) | 1.16 (1.11–1.21) | 1.17 (1.10–1.23) | 1.23 (1.17–1.29) |

Data are presented as mean (95% confidence interval)

**Supplementary Table 2. Characteristics of participants in each glycometabolic category in external validation.**

|  | Category 1 | Category 2 | Category 3 | Category 4 |
| --- | --- | --- | --- | --- |
| *n* | 213 | 135 | 32 | 72 |
| Sex (% women) | 0.48 | 0.49 | 0.38 | 0.47 |
| Age (years) | 38.6 (37.0–40.2) | 43.7 (42.0–45.5)* | 44.3 (40.6–47.9)* | 46.4 (44.2–48.6)* |
| Height (m) | 165.9 (164.7–167.1) | 165.4 (163.9–166.9) | 167.2 (164.4–170.1) | 165.2 (163.3–167.0) |
| BMI (kg/m^2^) | 22.0 (21.7–22.4) | 23.8 (23.2–24.4)* | 22.0 (21–23.1) | 23.9 (23.2–24.7)* |
| 30 mPG (mg/dL) | 124.3 (121.9–126.7) | 138.1 (135.8–140.4)* | 166.4 (163.5–169.4)* | 178.3 (173.6–182.9)* |
| 120 mPG (mg/dL) | 100.6 (98.5–102.8) | 142.0 (137.5–146.5)* | 100.4 (95.0–105.8) | 156.5 (148.9–164.2)* |
| Matsuda index | 10.7 (10.2–11.3) | 7.5 (6.7–8.2)* | 8.5 (7.1–9.8)* | 5.4 (4.7–6.2)* |

Data are presented as mean (95% confidence interval), percentage, or number of individuals. **p* < 0.05 vs. category 1. Abbreviations: BMI, body mass index; x mPG, x-min post-load plasma glucose level during the OGTT.

**Supplementary Table 3. Characteristics of the questionnaire answers for each glycometabolic category used in the external validation of the random forest model.**

|  | Category 1 | Category 2 | Category 3 | Category 4 |
| --- | --- | --- | --- | --- |
| Frequency of mobile phone and tablet computer use at bedtime | 2.28 (2.14–2.42) | 2.27 (2.09–2.46) | 2.59 (2.31–2.88) | 2.40 (2.14–2.66) |
| Do you wake up in the middle of the night? | 3.83 (3.69–3.96) | 3.84 (3.68–4.01) | 3.97 (3.68–4.26) | 3.74 (3.51–3.96) |
| Do you wake up late on non-working day? | 3.90 (3.77–4.03) | 3.98 (3.82–4.14) | 4.13 (3.76–4.49) | 4.01 (3.80–4.23) |
| Which do you usually eat: rice or bread? | 1.88 (1.73–2.03) | 1.72 (1.56–1.88) | 1.78 (1.34–2.22) | 1.63 (1.39–1.86) |
| Frequency of soup intake | 2.98 (2.79–3.17) | 3.05 (2.80–3.30) | 3.56 (3.13–3.99) | 3.43 (3.12–3.74) |
| Frequency of tea intake per week at lunch | 2.84 (2.57–3.10) | 2.95 (2.59–3.31) | 3.47 (2.78–4.16) | 3.33 (2.90–3.77) |
| Frequency of toothbrush replacement | 2.42 (2.28–2.57) | 2.30 (2.14–2.46) | 2.25 (1.91–2.59) | 2.51 (2.27–2.76) |

Data are presented as mean (95% confidence interval)
